# Revisiting Greek Propolis: Chromatographic Analysis and Antioxidant Activity Study

**DOI:** 10.1371/journal.pone.0170077

**Published:** 2017-01-19

**Authors:** Konstantinos M. Kasiotis, Pelagia Anastasiadou, Antonis Papadopoulos, Kyriaki Machera

**Affiliations:** 1 Benaki Phytopathological Institute, Department of Pesticides Control and Phytopharmacy, Laboratory of Pesticides’ Toxicology, Kifissia, Athens, Greece; 2 Benaki Phytopathological Institute, Department of Phytopathology, Laboratory of Non-Parasitic Diseases, Kifissia, Athens, Greece; Institute of medical research and medicinal plant studies, CAMEROON

## Abstract

Propolis is a bee product that has been extensively used in alternative medicine and recently has gained interest on a global scale as an essential ingredient of healthy foods and cosmetics. Propolis is also considered to improve human health and to prevent diseases such as inflammation, heart disease, diabetes and even cancer. However, the claimed effects are anticipated to be correlated to its chemical composition. Since propolis is a natural product, its composition is consequently expected to be variable depending on the local flora alignment. In this work, we present the development of a novel HPLC-PDA-ESI/MS targeted method, used to identify and quantify 59 phenolic compounds in Greek propolis hydroalcoholic extracts. Amongst them, nine phenolic compounds are herein reported for the first time in Greek propolis. Alongside GC-MS complementary analysis was employed, unveiling eight additional newly reported compounds. The antioxidant activity study of the propolis samples verified the potential of these extracts to effectively scavenge radicals, with the extract of Imathia region exhibiting comparable antioxidant activity to that of quercetin.

## Introduction

Propolis is a natural product that belongs to the great family of bee products. The word propolis is a complex term originating from two ancient Greek words: *pro*- standing for “before or in defense” and *polis* meaning city. Thus, in apiculture, its meaning refers to the harboring of the hive. Propolis is a sticky, resinous substance, collected from various floral sources that are transformed and used by honeybees to construct and maintain their hives by sealing holes in their honeycombs. It is also used for smoothing out the internal walls and shelter the entrance of the hive from intruders. Trends and development in propolis research have been reviewed by Bankova [1]. In this view, the essential point in any research conducted is the chemical variability of propolis attributed to the diversity of its plant origin [2]. Propolis is a traditional remedy in alternative medicine that has been used for centuries in Egypt, Greece, and other countries as well. Propolis possesses antimicrobial [3], anti-oxidative, anti-ulcer, immunomodulatory [4] and anti-tumor activities, and the latter is proved by a plethora of reports. An informative review article on the biological activity of bee propolis in health and disease was published by Lofty in 2006 [5], collecting a significant number of research results on various medicinal aspects.

Within this context, its biological activity is attributed to its chemical composition that encompasses mainly, phenolic compounds [6]. Plant polyphenols are known for their beneficial effect on health that is vastly described for oral health (indicatively see [7]). In this regard, many research groups have presented reviews and original reports on the beneficial effect on human health of many of its constituents [8–14]. In this context, some of its components have been shown to attenuate apoptosis in rat models (for pinocembrin see [15]). Caffeic acid phenethyl ester (CAPE) is an exemplary bioactive component of propolis, exhibiting a diversity of bioactivities, such as anti-tumor effects in pre-clinical models of human breast cancer or inhibition of growth of breast cancer stem cells [16,17]. In addition, many propolis components exist in some specific plant extracts as well and have been studied for their anti-cancer activity (see indicatively [18]). The chemical base of the biological activity of flavonoids, exemplified by their antioxidant properties, are collectively presented by Kancheva and Kasaikina [19].

Until recently, only three works have dealt exclusively with the phenolic composition of propolis extracts from Greece and Cyprus. Kalogeropoulos et al. used GC-MS analysis after derivatization reaction [3] to explore its chemical profile. An additional work was published by Graikou et al., using GC-MS again, to highlight the features of Meditteranean propolis, including samples from Greece, Cyprus, Croatia and Algeria [20]. The other was reported by Lagouri et al. in 2014, including a limited amount of compounds [21]. On international scale an indicative landmark targeted study was reported by Falcao et al., incorporating almost 40 analytes, that managed to efficiently display the chemical profile of Portuguese propolis [22].

Considering the importance of propolis due to its pharmacological properties, and the limited number of works on Greek propolis, we decided to revisit its chemical composition and assess the antioxidant activity of the studied extracts. The latter was reinforced by the demographics of the European agricultural industry that render Greece second regarding bee colonies number [23], designating a substantial potential for the exploitation of this matrix.

Therefore and considering that, to our knowledge, none targeted high-performance liquid chromatographic mass spectrometric (HPLC-MS) method analysis work on phenolic compounds of Greek propolis is reported, a multi-analyte HPLC-MS (using electronspray interface and diode array, HPLC-ESI-PDA/MS) method to monitor and quantify 59 compounds belonging to relevant bioactive chemical categories, such as aromatic acids and flavonoids, was developed. The selection of compounds included in the HPLC-PDA-ESI/MS method was based on previously published works on propolis, aiming to incorporate as many as possible compounds some of which were previously not described in Greek propolis. Furthermore, Artepillin C that is a unique constituent of Brazilian propolis was incorporated, although not expected to be detected. The method was applied to the analysis of eight propolis samples from Greece and to a Brazilian tincture propolis. Tentative characterization of new compounds *via* HPLC-PDA-ESI/MS under full scan mode was also pursued and reported. In addition, the use of GC-MS was implemented on a complementary basis, despite its extensive use in previous works. Last but not least, the propolis extracts were assessed for their antioxidant activity using a standard protocol. Such extracts, in previous works, have been evaluated extensively for their antioxidant activity exhibiting high radical scavenging activity (indicatively see [24]). Overall, both chromatographic methods revealed several new constituents, while one of the propolis extracts displayed comparative antioxidant activity to that of the bioactive molecule of quercetin. Finally, statistical analysis demonstrated certain correlations among the variables selected.

## Materials and Methods

### Chemicals and Reagents

The specific compounds, were: Caffeic acid, CAPE, chrysin, luteolin, daidzein, suberic acid, apigenin, (Alfa Aesar), pinocembrin, isorhamnetin, isosakuranetin, vitexin, orientin, rosmarinic acid, myricetin, vanillin, ursolic acid, hydroxytyrosol, tangeretin, chrysoeriol, betulinic acid, eriodictyol, sakuranetin, naringenin, *t*-cinnamic acid, genistein, diosmetin, resveratrol, galangin, pinocembrin 7-methyl ether, techtochrysin, (Extrasynthese) rutin, isoferulic acid, ellagic acid, kaempferol, quercetin, corosolic acid, acacetin, diosmin, protocatechuic acid ethyl ester, hesperetin, phloridzin, chlorogenic acid, *p*-coumaric acid, (±)catechin, maslinic acid, (Sigma Aldrich), rhamnetin, syringic acid, protocatechuic acid, ferulic acid, kaempferide, adipic acid, pinostrobin, gallic acid, pinobanksin (Fluka), naringin, hesperidin, (Acros Organics) pinobanksin-3*O*-acetate (Interchim Inc), cinnamylidenacetic acid, artepillin C (Wako Chemicals). o-Orselllinaldehyde was purchased from Santa Cruz Biotechnology (USA).

Water, acetonitrile, methanol and formic acid were purchased from Fisher Scientific, UK and they were of LC-MS grade. Ethanol was purchased from Merck, Germany. PTFE filters (0.45 μm) were obtained from Macherey-Nagel, Germany.

### Samples

The propolis samples were obtained directly from eight individual beekeepers from several regions/locations of Greece. Nafplio (Argolida, Eastern Peloponnese), Amorgos (Cyclades Islands, Aegean Sea), Crete (Heraklion, Central Crete), Kos (Dodecanese Islands, South Aegean), Lakonia (Areopoli, Southern Peloponnese), Imathia (Central Macedonia, North Greece), Arkadia (Central Peloponnese) and Corfu island (Ionian Sea). The samples were obtained after the honey-harvesting season, by scraping as indicated by the local beekeepers. The propolis samples were collected during 2014–2015, and they were stored at -20°C before extraction. Finally, a Brazilian green propolis tincture (commercially available) was obtained and analyzed to verify method’s ability to detect and quantify, Artepillin C, its most bioactive component, which is unique for Brazilian propolis.

### Preparation of Extracts

Raw, crude propolis was cut into very small pieces and was homogenized. Then, 10 mL of a 4:1 mixture solution of ethanol-water respectively, was added to 1 g of the propolis, and the mixture was continuously stirred for 24 h at room temperature, in dark. Subsequently, the crude mixture was transferred to a falcon tube and subjected to centrifugation (Heraeus Labofuge 400R Thermo Electron Corporation, 5 min, 10°C, and 4000 rpm). The upper liquid layer was then decanted, filtered, and stored at -20°C overnight. The latter favored the removal of waxes (due to their precipitation). The above procedure was repeated to ensure quantitative extraction of compounds. The resulting combined solution was filtered, evaporated to near dryness and then freeze-dried. The derived dry extract was reconstituted in a 4:1 ethanol-water solution. Before injection into the HPLC-ESI-PDA/MS system, the extract was diluted with methanol to avoid contamination of the mass spectrometer and concentrations to fall within the calibration curve range, and finally filtrated using PTFE filter. For GC-MS analysis pure EtOH was used as extraction medium, and the duration of extraction was lowered to 5 h. EtOAc extraction was also applied for 5 h, with not substantial differences in terms of compounds extracted compared to EtOH.

### High-Performance Liquid Chromatography-Electrospray Photo Diode Array Mass Spectrometry

A Shimadzu (Kyoto, Japan) LCMS-2010 EV Liquid Chromatograph Mass Spectrometer instrument was used with the LCMS solution version 3.0 software consisting of an SIL-20A prominence autosampler and an SPD-M20A diode array detector. The latter were coupled in series with a mass selective detector equipped with an atmospheric pressure ionization. The LC separation was achieved on a Zorbax Eclipse Plus, 3.5 μm, 150 × 2.6 mm i.d. chromatographic column. The mobile phase consisted of two channels, channel (A) 0.1% formic acid in water (A) and channel (B) pure acetonitrile (B). The flow rate was set at 0.3 mL•min^-1^ and the column gradient program started at 20% B, and ramped linearly over the course of 10 min at 30% B. Subsequently the system was linearly increased until the 40 min to 40% B, where it was maintained up to the 70^th^ min. Then, the acetonitrile percentage was linearly increased over 25 min to 70%, and kept for additional 5 min. Then, acetonitrile returned in the course of 5 min at the initial concentration of 20%, where it stayed for additional 3 min. Overall runtime was extended to 108 min, albeit the majority of compounds elute before the 50^th^ minute. Electron Spray Ionization (ESI) mode (using discrete events for each analyte monitored) was utilized, functioning in the selected ion monitoring mode (SIM). Photodiode array monitored wavelengths from 190 to 800 nm.

### Validation of the Present Method—Quantification of Constituents

The developed method was validated following mainly the International Conference on Harmonization [25] also considering the SANCO document [26]. A review publication, pertinent to chemical measurements in natural products research was also regarded [27]. Validation study was performed regarding recovery, linearity, intra-day and inter-day precision. The calibration curves were established using the dilute standard solution of the 59 compounds of the method. Calibration curves ranged from 40 to 5000 ng/mL. The selected range was decided considering three parameters: a) the coverage of expected concentrations usually encountered for these compounds after extraction, sample preparation and dilution, b) the MS detector protection from high concentration levels and c) the analytical performance of the method. Blank experiments were also conducted (complete procedure without matrix extract). Standard addition was used for the recovery study at two concentration levels.

In this context, the precision of the chromatographic method was expressed as the RSD % of the repeatability (intra-day) and intermediate precision (inter-day) analyses (*n* = 3) over 1, 2 and 3 days. Repeatability and intermediate precision were considered acceptable when relative standard deviation values (RSD%) were < 20%. LOQs were defined as the lowest validated spiked level that met the method performance acceptability criteria, regarding mean recoveries in the range of 70–120%, with RSD_r_ 20%.

To estimate if the matrix impacts considerably the peak area and, therefore, the sensitivity of the analytes, the slopes of the calibration lines obtained for propolis after standard addition (b_matrix_) and the solvent (b_solvent_) were divided to determine the matrix factor and the % matrix effect (ME) was calculated by Eq (1).

$$\text{\%ME}=\left(1-\frac{{\text{b}}_{\text{matrix}}}{{\text{b}}_{\text{solvent}}}\right)\times 100.$$ (1)

### GC-MS Conditions

The GC-MS analysis was performed on a Chromtech Evolution MS/MS triple quadrupole mass spectrometer built on an Agilent 5975 B inert XL EI/CI MSD system that was operated in full scan data acquisition mode. Samples were injected with a Gerstel MPS-2 autosampler using a 10-μL syringe. Separations were performed on an HP-5ms UI, length 30m, ID 0.25mm, film thick. 0.25 μm (J&W Folsom, USA). Helium was used as the carrier gas at a flow rate of 1.2 mL min^-1^. The column oven temperature program started from 80°C, staying for 3 min, increased to 160°C at a rate of 8°C min^-1^ where it remained for 10 min, then increased to 220°C at a rate of 13°C min^-1^ and held for 5 min. Then the temperature was raised to 260°C min^-1^ at a rate of 5°C min^-1^, held for 10 min and finally ramped to 300°C at a rate of 5°C min^-1^ held for 2 min. The transfer line, manifold, and source of ionization temperatures were 300, 40 and 230°C. The electron multiplier voltage was set at 2000 V. The total GC analysis lasted for 56.62 min.

Identified peaks in GC-MS were confirmed by comparing the acquired mass spectra with those in the commercial library of NIST 08.

### DPPH Radical Scavenging Assay

The selected concentrations of the ethanolic extracts were assessed in the range of 0.05 to 250 μg/mL. The latter was established after initial testing of indicative levels so as to determine the final work range depending on the % antioxidant activity. Sample stock solutions of 1 mg/mL were diluted to the final concentration of ethanol. A 0.3 mM DPPH ethanol solution was prepared in absolute ethanol. The experimental part consisted of mixing of 1 mL of DPPH solution with 2.5 mL of the propolis extract [28, 29]. Then, after a gentle stirring of 1 min, the solution was left for 30 min at room temperature so as the extract to react with DPPH and scavenge it. The absorbance was measured at 520 nm, in a spectrophotometer (Shimadzu, UV-VIS, Pharma-Spec 1700). Ethanol was utilized as a negative control while quercetin was used as positive control. Control was composed of ethanol (2.5 mL) and DPPH radical solution (1 mL). All the analyses were carried out in triplicate.

### Total Phenolic Content

Folin Ciocalteau is a mixture of phosphomolybdate and phosphotungstate utilized for the colorimetric *in vitro* assay of phenolic and polyphenolic antioxidants [30,31]. In this context, it was used for the determination of total phenolic content (TPC). Briefly, 20 μL of the propolis extract (1 mg/mL) were sequentially mixed with 300 μL of distilled water and 100 μL of Folin Ciocalteau’s phenol reagent. After 4 min, 1000 μL of distilled water and 400 μL of 20% sodium carbonate were added. The reaction mixture was kept in the dark for 2 h at room temperature and absorbance was measured at 760 nm in a spectrophotometer (Shimadzu, UV-VIS, Pharma-Spec 1700). TPC was calculated from the calibration curve generated from standard solutions of gallic acid ranging from 0.5 to 20 μg/mL (y = 0.0082x, r^2^ = 0.9980, intercept = 0.0018). The latter was expressed as gallic acid equivalent (mg) per gram of extract (mg GAE/g _dry extract_). Blank was prepared as above using ethanol instead of propolis extract. All analyses were performed using three aliquots of each extract sample, measured in triplicate, calculating the average value.

### Total Flavonoids Content

Total flavonoid contents (TFC) were determined by the aluminium colorimetric method [32], using quercetin as reference standard [33]. More specifically, in an aliquot (100 μL) of propolis extract (1 mg mL^-1^) was sequentially added 1 mL of methanol, 3.5 mL of HPLC water, 2% (w/v) AlCl_3_ (200 μL) and 1 M potassium acetate (200 μL). After 30 min of incubation at room temperature, the absorbance was measured at 435 nm by a spectrophotometer. The TFC was expressed as μg of quercetin equivalent per mg of dry weight of the extract. All analyses were performed using three aliquots of each extract sample, measured in triplicate, calculating the average value.

### Principal Component Analysis

The analysis was performed with SPSS 20.0 (IBM Corp.). All data were tested as for whether they were normally distributed by the Shapiro-Wilk’s test and the visual inspection of data’s histograms, normal Q-Q plots and box plots. Further, data were tested for equality of variances (homoscedasticity) by the Levene’s test. For those samples, which failed to comply with the conditions of normality and homoscedasticity, the logarithmic transformation log(x+1) was applied.

For the DPPH derived IC_50_ values, lower values reflect higher antioxidant activity. Therefore, for the PCA purpose, all values were normalized by setting the lowest IC_50_ value as 100, and rest of values were adjusted accordingly.

## Results

### Chromatographic Separation

**Fig 1:**
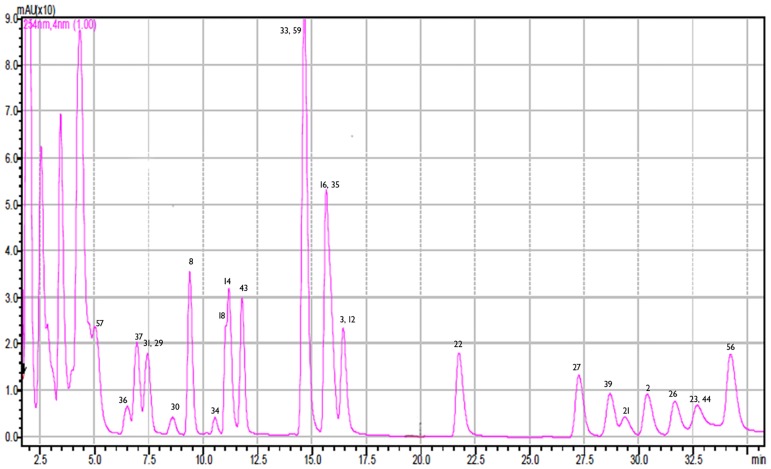
Magnified HPLC-UV chromatogram of a standard solution (500 ng/mL), at 254 nm.

**Fig 2:**
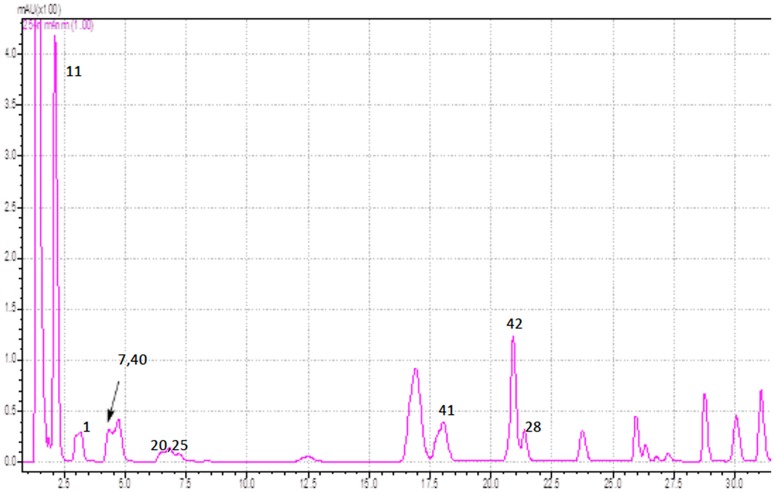
HPLC-UV chromatogram (at 254 nm) of standard solution mix (500 ng/mL) with delayed elution of compounds (indicative marking).

The developed HPLC-PDA-ESI/MS method managed to separate with substantial resolution the majority of targeted analytes (see Fig 1). Considering that quantitative analysis was performed predominantly using the SIM mode that monitors few mass to charge values (m/z) depicted in Table 1, and the background is reduced, the efficient separation of all analytes amongst them was not a prerequisite. Nevertheless, to provide better resolution for compounds eluting from 1.5 min to 2.1 min a modification of the mobile phase was implemented. Hence, an increase of water in the mobile phase resulted in delayed elution and enhanced resolution especially for the compounds eluting between 1.5–2 min (see Fig 2).

**Table 1:** Characterization of phenolic compounds by HPLC-DAD-ESI/MS.

Compound Number	Compound Name	t_R_ (min)	λ_max_ (nm)	Quantitation ion	Confirmation ion(s)	Mode polarity	Voltage (kV)	Event N°
1	Protocatechuic acid	2.1	260, 294	153	109	ESI(-)	2.1	Event 1
2	Pinocembrin	30.4	289	255	213	ESI(-)	1.6	Event 2
3	Kaempferol	16.3	264, 366	285	151	ESI(-)	2.1	Event 3
4	Apigenin	15.3	268, 337	269	225, 151	ESI(-)	1.8	Event 4
5	Chrysin	28.2	268	253	209	ESI(-)	1.6	Event 5
6	Galangin	31.4	265, 356	269	241, 227	ESI(-)	1.6	Event 6
7	Chlorogenic acid	2.1	325	353	191	ESI(-)	2.0	Event 7
8	Daidzein	9.5	255	253	208	ESI(-)	2.1	Event 8
9	Ellagic acid	3.6	252, 367	301	145	ESI(-)	1.8	Event 9
10	Ferulic acid	4.6	295	193	134	ESI(-)	2.0	Event 10
11	Gallic acid	1.8	280	169	125	ESI(-)	2.1	Event 11
12	Hesperetin	16.4	340	300.8	163.9	ESI(-)	1.9	Event 12
13	Hydroxytyrosol	2.0	277	153	-	ESI(-)	2.3	Event 13
14	Luteolin	11.7	268, 349	285	241	ESI(-)	2.1	Event 14
15	p-Coumaric acid	3.9	309	163	119	ESI(-)	2.4	Event 15
16	Pinobanksin	15.4	291, 330	271	253, 225	ESI(-)	2.4	Event 16
17	PIN-7ME	57.7	286	271	270	ESI(+)	1.6	Event 17
18	Quercetin	11.6	255, 370	301	151	ESI(-)	1.8	Event 18
19	Tectochrysin	53.2	268	269	226	ESI(+)	2.2	Event 19
20	Caffeic acid	2.5	291, 321	179	135	ESI(-)	1.6	Event 20
21	Sakuranetin	29.6	284	285	286	ESI(-)	2.4	Event 21
22	Rhamnetin	22.1	255, 367	315	300, 193	ESI(-)	1.8	Event 22
23	CAPE	33.5	245, 298, 325	283	179, 135	ESI(-)	1.8	Event 23
24	Pinostrobin	66.9	289	271	167, 131	ESI(+)	2.3	Event 24
25	Syringic acid	2.0	273, 217	197	182	ESI(-)	2.6	Event 25
26	Kaempferide	32.5	335, 265	299	284, 151	ESI(-)	2.0	Event 26
27	Acacetin	27.8	268	283	269	ESI(-)	2.0	Event 27
28	Rutin	2.7	255, 354	609	301, 273	ESI(-)	2.0	Event 28
29	Protocatechuic acid ethyl ester	7.3	260, 222, 294	181	182, 153	ESI(-)	2.0	Event 29
30	Resveratrol	8.5	305, 216	227	184.9	ESI(-)	2.0	Event 30
31	Phloridzin	7.5	282, 234	435	273	ESI(-)	2.0	Event 31
32	Maslinic acid	76.6	277	471	453, 425	ESI(-)	2.1	Event 32
33	Naringenin	14.6	288, 233	271	119	ESI(-)	1.9	Event 33
34	Eriodictyol	10.7	287, 233	287	151	ESI(-)	2.1	Event 34
35	Diosmetin	15.7	347, 246	299	284, 256	ESI(-)	2.0	Event 35
36	Rosmarinic acid	6.6	327, 236	359	161	ESI(-)	2.5	Event 36
37	Myricetin	6.8	372, 253	317	178.9	ESI(-)	1.9	Event 37
38	Isorhamnetin	16.5	371, 254	315	301	ESI(-)	2.0	Event 38
39	Isosakuranetin	29.2	288, 234	285	178.9	ESI(-)	2.0	Event 39
40	(+)-Catechin	1.6	280	291	-	ESI(+)	2.5	Event 40
41	Orientin	2.5	348, 254	447	-	ESI(-)	2.0	Event 41
42	Vitexin	2.8	360, 268	431	283	ESI(-)	1.5	Event 42
43	*trans*-Cinnamic acid	12.1	276	149	-	ESI(+)	2.0	Event 43
44	Pinobanksin 3-*O*-acetate	33.0	280	313	253	ESI(-)	2.2	Event 44
45	Cinnamylideneacetic acid	71.2	229, 276	219	-	ESI(-)	2.4	Event 45
46	Artepillin C	63.2	315	299	255	ESI(-)	2.1	Event 46
47	Adipic acid	2.9	210	145	126.8, 100.9	ESI(-)	2.3	Event 47
48	Ursolic acid	100.4	215	439	411, 457	ESI(+)	2.3	Event 48
49	Suberic acid	3.2	-	173.1	-	ESI(-)	2.5	Event 49
50	Genistein	14.2	260	269	133	ESI(-)	2.1	Event 50
51	Hesperidin	5.1	281, 229	609	-	ESI(-)	2.2	Event 51
52	Corosolic acid	77.0	-	471.4	425	ESI(-)	2.2	Event 52
53	Betulinic acid	97.3	-	455.5	-	ESI(-)	2.0	Event 53
54	Isoferulic acid	4.8	323, 240	195	177	ESI(+)	2.4	Event 54
55	Naringin	4.9	283	579	-	ESI(-)	1.4	Event 55
56	Tangeretin	34.4	325, 270	373.1	343.3	ESI(+)	1.8	Event 56
57	Diosmin	5.3	345, 253	607	-	ESI(+)	1.8	Event 57
58	Vanillin	4.1	230, 279	153	-	ESI(+)	2.5	Event 58
59	Chrysoeriol	15.1	348, 254	298.9	283.9	ESI(-)	2.2	Event 59

### Identification-Confirmation of Compounds

Identification and confirmation of compounds was achieved by comparing the m/z values, the retention time and UV absorption spectrum with those of the analytical standards, also considering characteristic works of the relative literature [22,34,35]. ESI ionization proved substantial in producing characteristic ions for the compounds studied (see also “Figures E and F in S1 File” and “Figures A-Z, Figures A1-Z1, and Figures A2-C2 in S2 File”). Peak purity index in the UV-spectra, as it was produced by the respective LC software (see Table 1 for maximum wavelengths obtained for each compound) assisted selectivity evaluation, regarding as pure, the peaks with spectra similarity exceeding 95%. It is noteworthy, however, that in natural products hundreds of compounds not listed in targeted analysis, can potentially co-elute with listed compounds. Therefore, caution is required in quantitative analysis. This “risk” is minimized when MS is used in line with UV, as in the presented work.

### Method Validation Results

With regard to method validation characteristics, these are presented in Table 2. Linearity was checked for all analytes in the ranges of 40–5000, and 60–5000 ng/mL with acceptable correlation coefficient values (r^2^ ≥ 0.99). Recovery was assessed at two levels by standard addition of the standards mix solutions to the samples (in triplicate), and it was above 70%. LOQs were determined at 40 and 60 ng/g_extract_ for two sets of compounds as presented in Table 2, fulfilling the respective criteria (as mentioned above). Concerning matrix effect, slight signal suppression was evidenced for the majority of analytes (see Table 3 for ME results). However, the %ME, never surpassed the threshold value of ±20%, which is considered low based on proposed literature classification [36].

**Table 2:** Analytical Method Validation Characteristics.

				**Calibration Range 40–5000 (ng/mL)**			
**Analyte**	**Regression Equation**	**Regression coefficient (R**^**2**^**)**	ME (%)	Recovery ±RSD %		Inter-d precision	Intra-d-precision
				n = 3		RSD % n = 3	
				100 ng/g	1000 ng/g	100 ng/g	100 ng/g
Apigenin	y = 17118x+735090	0.9962	-2.2	82±10	77±15	3.40	3.02
Chrysin	y = 4074,4x-14955	0.9991	-1.9	94±10	93±14	1.05	1.65
Galangin	y = 3603,9x-5504,1	0.9993	-0.8	90±15	87±9	3.30	2.56
Ellagic acid	y = 4181,8x+459836	0.9900	-3.8	85±11	87±13	2.18	4.02
Ferulic acid	y = 5287x+461871	0.9947	-5.2	81±14	73±9	3.05	5.04
Hesperetin	y = 7799,5x+407655	0.9945	-1.1	83±10	90±12	1.09	1.95
Luteolin	y = 103735x-4000000	0.9926	-0.9	78±11	80±8	3.78	3.14
p-Coumaric acid	y = 6176,9x+209484	0.9985	-4.4	73±5	79±6	2.89	2.49
Pinobanksin	y = 8394,7x+299561	0.9970	-6.9	79±10	76±7	1.67	1.77
Quercetin	y = 5472,7x-28037	0.9993	-7.2	93±12	85±9	5.39	4.72
Caffeic acid	y = 1729,9x-63292	0.9968	-0.6	69±10	69±7	2.84	2.94
CAPE	y = 24466x+782287	0.9951	-8.1	91±13	88±10	4.04	4.89
Rhamnetin	y = 13067x-578824	0.9959	-2.3	80±14	74±5	5.40	5.15
Kaempferol	y = 60404x-593055	0.9976	-1.8	78±6	84±7	0.80	2.42
Chlorogenic acid	y = 26764x-709939	0.9995	-7.7	76±7	80±10	3.02	5.25
Protocatechuic acid	y = 437,1x+21073	0.9992	-0.9	80±14	88±16	2.47	2.85
Syringic acid	y = 3901,6x-18827	0.9991	-2.3	80±12	77±6	3.17	6.01
Daidzein	y = 63929x+3000000	0.9929	1.2	80±12	86±5	1.44	1.65
Kaempferide	y = 23465x-123085	0.9919	0.4	79±13	82±12	1.28	2.34
Acacetin	y = 9665,1x+603921	0.9933	-8.3	88±7	79±9	3.54	2.91
Resveratrol	y = 102223x-632009	0.9988	-2.9	81±7	79±10	7.01	6.72
Naringenin	y = 6670,4x+39019	0.9928	-8.1	85±12	91±14	8.11	4.38
Adipic acid	y = 8841,6x-42008	0.9972	-7.5	89±10	82±14	6.21	3.26
Betulinic acid	y = 44586X-311102	0.9956	-4.0	89±15	79±5	1.98	3.29
Cinnamylidene acetic acid	y = 22654x-188755	0.9991	-2.9	95±10	89±5	1.17	1.29
Pinobanksin 3*O*-acetate	y = 1333,7x-7987	0.9972	-6.9	77±3	83±4	3.23	3.04
Vitexin	y = 40983x-283008	0.9987	0.8	95±7	83±4	1.01	3.24
Orientin	y = 41202x+326081	0.9919	0.9	85±7	81±10	4.45	3.74
Isosakuranetin	y = 5565,7x-200093	0.9966	-3.6	91±14	81±6	4.05	2.91
Myricetin	y = 11123x-509882	0.9933	-1.3	91±7	84±9	6.01	2.31
Rosrmarinic acid	y = 203347x-550899	0.9944	-1.4	83±7	84±12	4.11	2.78
Genistein	y = 1009,8x-16789	0.9992	-8.4	78±5	92±13	5.21	2.44
Tangeretin	y = 37768,4x-309887	0.9982	-1.1	83±7	88±9	1.12	3.01
Diosmin	y = 15006χ+201910	0.9963	-2.7	92±11	86±8	2.23	3.71
Diosmetin	y = 4571,1χ-16730	0.9909	-1.9	92±7	79±9	1.08	1.99
Isorhamnetin	y = 55601x+320985	0.9948	0.7	80±5	80±8	1.29	3.61
Hydroxytyrosol	y = 4201,8x-89002	0.9917	-4.9	91±9	80±11	4.04	2.17
Vanillin	y = 16723x-236489	0.9917	1.1	83±13	90±14	1.10	1.38
				**Calibration Range 60–5000 (ng/mL)**			
				**Recovery ±RSD %**		Inter-d precision	Intra-d-precision
				n = 3		RSD % n = 3	
				100 ng/g	1000 ng/g	100 ng/g	100 ng/g
Gallic acid	y = 1496,8x-63708	0.9972	1.3	84±7	83±10	2.94	3.47
Pinocembrin	y = 3049,9x-44337	0.9960	-0.8	83±11	87±9	2.20	2.85
PIN-7ME	y = 329,7x+21307	0.9997	-2.2	84±10	79±9	5.39	4.97
Pinostrobin	y = 33166x+4000000	0.9976	-6.8	91±7	85±12	1.55	1.82
Tectochrysin	y = 1300,6x-16230,5	0.9999	-3.5	81±10	77±6	5.26	4.82
Sakuranetin	y = 1240,2x-27612	0.9986	-3.2	91±9	88±8	1.06	3.76
Rutin	y = 875,5x+11099	0.9984	-0.8	80±3	85±9	2.78	2.98
Maslinic acid	y = 3210,1x-88702	0.9947	0.5	77±8	87±12	5.02	6.03
Phloridzin	y = 7745,1x+30222	0.9990	-2.7	91±3	82±8	2.11	1.98
Artepillin C	y = 15557,1x-409876	0.9972	-4.3	75±3	88±11	4.98	6.04
Ursolic acid	y = 1349,8χ-20778	0.9991	-8.2	79±7	82±14	3.71	5.11
Suberic acid	y = 45520x+239044	0.9909	1.2	92±15	87±8	1.92	3.02
Hesperidin	y = 6609,5x-101009	0.9918	-3.9	90±11	83±6	3.47	2.19
Isoferulic acid	y = 980,5x-17750	0.9992	0.6	91±9	82±12	3.04	5.19
Corosolic acid	y = 30004x+220887	0.9990	0.4	81±10	79±5	1.55	4.92
Eriodictyol	y = 60430x-390334	0.9973	-2.5	101±11	89±7	3.01	2.84
Chrysoeriol	y = 3090,5x+23098	0.9992	-3.0	85±4	91±8	4.12	6.71
Naringin	y = 52234x+144502	0.9994	1.0	79±11	78±8	4.12	6.71

**Table 3:** Quantitation of constituents in Greek propolis extracts.

	Content μg/g (dry extract), n = 3							
Analyte	Arkadia	Kerkira	Nafplio	Amorgos	Crete	Kos	Lakonia	Imathia
Pinocembrin	1710.5	581.9	4381	361.2	3560	3944	1170.5	13992
Apigenin	120.3	nd	40.5	nd	78.8	939.7	240.8	1989.8
Chrysin	3490.6	1011.4	3790.2	246.1	169.7	1609	825.2	9940.3
Galangin	22.5	nd	1501	22.4	154.2	2589	756.3	2529.1
Ellagic acid	nd	39.8	nd	nd	nd	nd	nd	49.7
Tectochrysin	49.5	nd	nd	nd	nd	nd	nd	55.4
Syringic acid	9.3	nd	nd	nd	7.5	nd	nd	nd
Ferullic acid	61.8	73.9	nd	nd	nd	17.4	2.8	nd
Gallic acid	47.6	nd	263.2	44.9	33.2	177.1	61.0	27.9
Hesperetin	nd	nd	nd	nd	19.9	55.8	nd	nd
Luteolin	23.4	nd	336	25.6	nd	41.1	248.1	206.3
p-Coumaric acid	186.8	nd	846	36.4	52.5	1147	50.1	186.8
Pinobanksin	82.1	nd	130.1	nd	229.7	1235	326.9	190.0
PIN-7ME	270.9	nd	1018	280.3	nd	nd	nd	260.6
Caffeic acid	34.4	nd	nd	138.1	135.3	1673	228.3	nd
Pinostrobin	28.1	nd	nd	nd	nd	nd	679	nd
CAPE	37.7	nd	39.4	402.4	133.7	310.9	85.8	nd
Quercetin	43.2	nd	596.5	318.6	nd	nd	201.1	nd
Rhamnetin	39.3	nd	46.1	nd	nd	nd	89.1	nd
Kaempferol	nd	92.5	38.0	nd	260.7	179.6	1343	89.3
Chlorogenic acid	nd	nd	301.6	23.8	124.6	28.5	nd	61.1
Protocatechuic acid	nd	nd	nd	29.2	102.4	99.5	36.1	97
Kaempferide	nd	nd	3.2	nd	nd	nd	nd	nd
Acacetin	nd	nd	nd	96.3	226.4	nd	354.3	557.5
Resveratrol	nd	nd	0.9	nd	nd	nd	nd	1.4
Eriodictyol	34.1	nd	nd	nd	nd	932.7	nd	992.6
Naringenin	392.5	nd	721.7	302.9	nd	nd	1027	nd
Pinobanksin-3*o*-acetate	742	nd	nd	2949	nd	nd	1870.3	2809
(+)-Catechin	1641	nd	nd	nd	nd	297.7	1925	2717
Rutin	346.4	63.7	nd	nd	nd	237.5	nd	nd
Isorhamnetin	nd	nd	931	nd	nd	nd	257.3	151.4
Sakuranetin	nd	nd	nd	nd	nd	nd	nd	727
Isosakuranetin	nd	nd	nd	nd	76.7	nd	134	nd
Daidzein	nd	nd	nd	nd	nd	26.3	nd	nd
Vitexin	nd	nd	nd	nd	nd	nd	nd	30.3
Rosmarinic acid	nd	58.3	nd	nd	nd	nd	nd	nd
Myricetin	nd	nd	nd	nd	119	nd	479.2	nd
Ursolic acid	nd	nd	nd	nd	nd	nd	369.1	nd
Genistein	182.8	nd	nd	nd	nd	420.3	nd	nd
Cinnamilidene acetic acid	nd	nd	nd	nd	nd	nd	41.2	nd
*t*-Cinnamic acid	1.8	132.6	nd	nd	71.7	nd	nd	217.7
Vanillin	nd	101.7	nd	83.9	nd	nd	nd	nd

± standard deviation (SD) was less than 10%, nd, non detected

### Quantitation of Constituents

**Fig 3:**
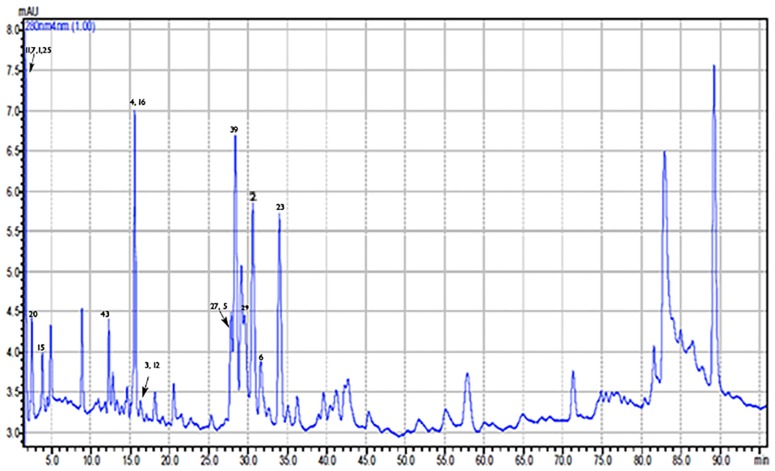
HPLC-UV chromatogram of Crete propolis extract at 280 nm.

**Fig 4:**
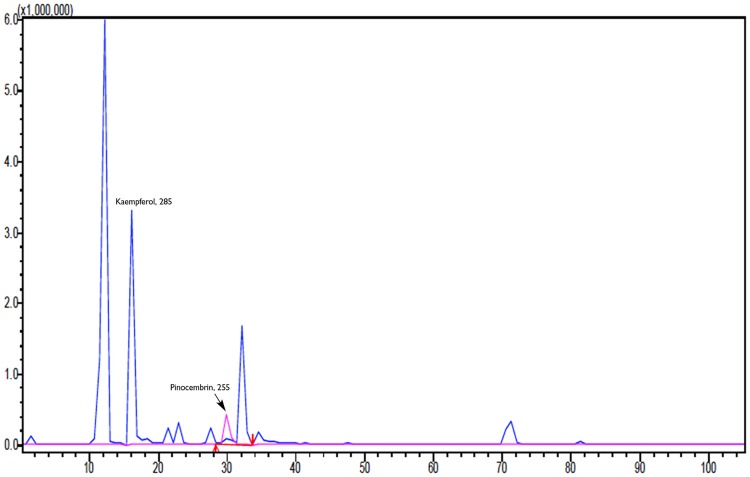
SIM chromatogram showing quantitation ions for kaempferol and pinocembrin (Crete sample).

**Fig 5:**
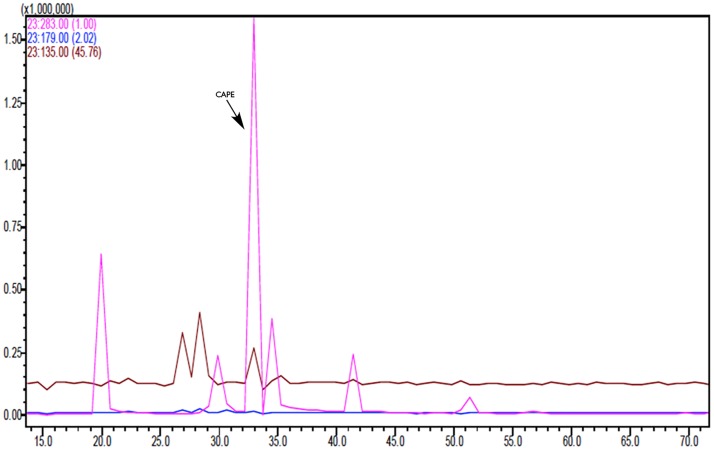
SIM chromatogram showing quantitation and confirmation ions for CAPE (Crete sample).

**Fig 6:**
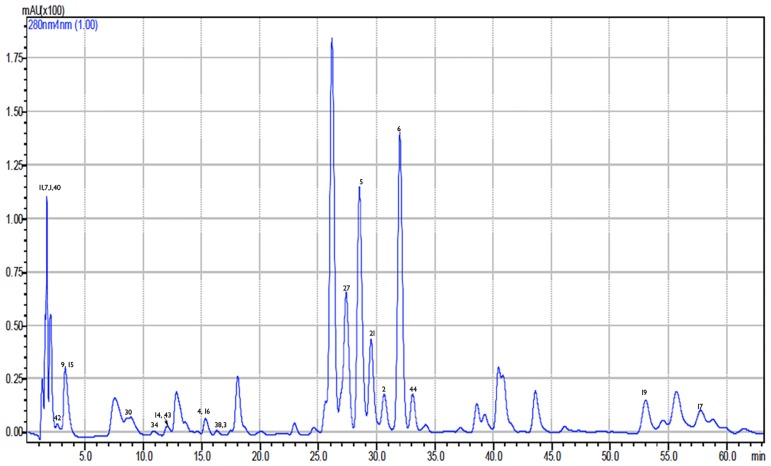
Magnified HPLC-UV chromatogram (at 280 nm) of Imathia propolis extract.

**Fig 7:**
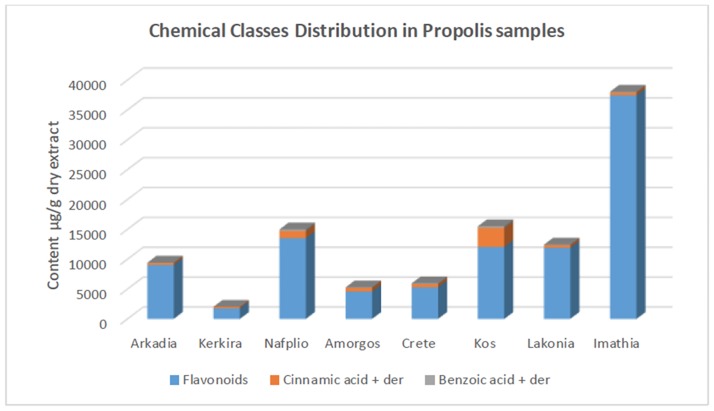
Distribution of chemical classes of compounds in propolis of different Greek regions.

**Fig 8:**
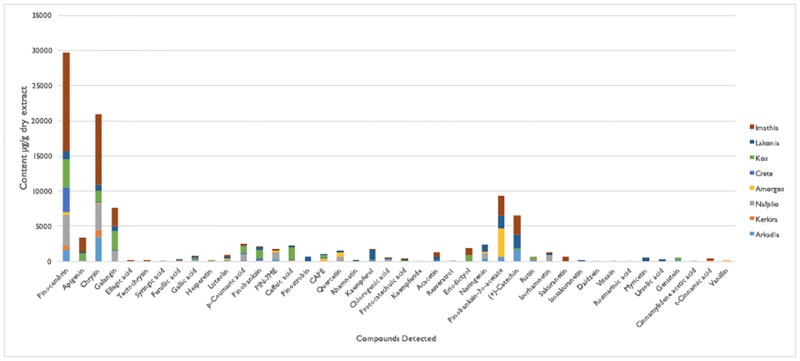
Distribution of individual compounds in propolis of different Greek regions.

The analysis of eight Greek propolis samples from Crete, Kos, Kerkira and Amorgos islands, Arkadia, Lakonia, Imathia, and Nafplio regions revealed the presence of several phenolic compounds (indicatively for Crete see Figs 3, 4 and 5, for Imathia Fig 6, and representative chromatograms in supplementary material “Figures C-D in S1 File”). Amongst them, various new constituents, namely isosakuranetin, luteolin, rhamnetin, hesperetin, acacetin, kaempferide, eriodictyol, and pinostrobin were detected. All the above components are reported for the first time in analyzed Greek propolis samples. Quantitative results for each region are depicted in Table 3, while in Figs 7 and 8 the distribution of components is portrayed (both as chemical classes and as individual compounds). Quantitation was performed with the HPLC-ESI/MS methodology; results, however, were verified with UV as well (particularly for the most abundant compounds). In this context, the six most dominant compounds for all regions were pinocembrin, chrysin, galangin, apigenin, pinobanksin 3-*O*-acetate, and (±)catechin that is in accordance with literature for European propolis major polyphenolic constituents (indicatively see [3,22, 34,37]).

The results demonstrate that propolis sample from Imathia was of the most diverse regarding compounds identified and contained the highest concentrations (higher than the other studied samples).

### Full Scan MS and PDA Putative Identification

Mass spectrometry data were also acquired in full scan mode (both negative and positive mode). Due to the chemistry of phenolic compounds, negative full scan mode was the most decisive tool in revealing the putative presence of several compounds not listed in the targeted screening approach. Several compounds were identified and are depicted in the respective Table (see Supplementary Material, “Table A in S1 File,”). To proceed to the tentative identification of substances, the literature on chemical constituents reported for propolis was considered. In particular, characteristic mass spectrometry ions-fragments for described compounds in the bibliography [22,34] were collected and compared to the fragment ions obtained in the current analyses. Elution order considering the chemical structures and subsequent polarity, and the characteristics of the chromatographic column used, have also been taken into account and paralleled to the bibliographical data.

### GC-MS Analysis

Primary criterion in GC-MS analysis using full scan mode, is the computed match factor of the studied spectrum and the respective one of the library. The estimated non-polar retention index (n-alkane scale) was also used in parallel with respective literature values where applicable, and appear in Table 4. It is noteworthy that some compounds identified by HPLC-ESI-PDA/MS were also confirmed by GC-MS. Indicative examples are pinocembrin, naringenin, chrysin, techtochrysin, and others. The latter is not a “surprising finding” since GC analysis of those and similar constituents, has been reported by Christov and Bankova in 1992, using capillary GC and GC-ECD [38,39].

**Table 4:** Distribution of newly identified compounds (assessed by GC-MS) in ethanolic extracts of Greek propolis.

RI Reference	RI_L_	RI_E_*	Retention Time (min)	Analyte	Nafplio	Amorgos	Crete	Kos	Imathia
-	no	1536	12.22	o-Orsellinaldehyde**					X
-	no	1564	19.91	Cinnamylidene acetic acid					X
[46]	1969	1717	22.40	Eudesmic acid		X			
-	no	1694	24.82	3,8-dimethyl-4-(1-methylethylidene)-2,4,6,7,8,8a-hexahydro-5(1H)-azulenone		X			
[47]	2700	2705	42.03	Heptacosane	X				
[47]	2800	2804	46.88	Octacosane	X				
-	no	2797	54.07	(+)-Episesamin			X		
-	no	3047	56.15	1-Octacosanol				X	

* RI_E_ Estimated non-polar retention index (n-alkane scale), **RI**_**L**_ literature non-polar retention index

** Confirmed by analytical standard

**Fig 9:**
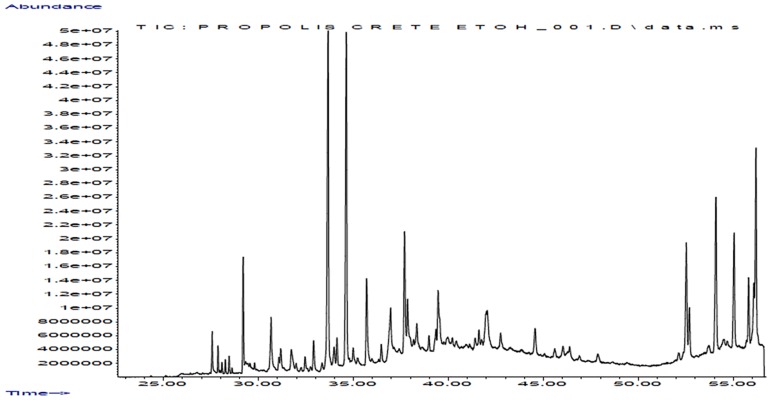
Magnified GC-MS chromatogram of Crete propolis sample.

Several of the identified constituents such as cedrol, epicedrol, ferruginol, α-cadinol, have been reported as constituents of Greek propolis by several research groups (indicatively see [40]). Since, in this work, GC-MS analysis aimed only at the identification of new compounds the overall GC-MS fingerprint is not reported (however an obtained GC-MS chromatogram is presented, see above Fig 9, accompanied by indicative GC-MS mass spectra, “Figures A and B in S1 File”). GC-MS analysis of the samples revealed the existence of additional compounds, to our knowledge, not previously reported in Greek propolis samples (see the list of compounds in Table 4).

Indicatively, first report of 3,8-dimethyl-4-(1-methylethylidene)-2,4,6,7,8,8a-hexahydro-5(1H)-azulenone is herein made (see Table 4, Amorgos extract). Azulene type compounds in Greek propolis have been reported only by Kalogeropoulos et al., namely, 1,2,3-triphenyl azulene was detected. Eudesmic acid found in Amorgos sample is an O-methylated trihydroxybenzoic acid that is reported in *Eucalyptus* spp. extracts [41]. 1-docosene, hexadecane, octacosane, hexacosene and heptacosane identified in Nafplio sample, are also reported for the first time in Greek propolis. Octacosane was reported as a constituent of Dubai propolis [42], while heptacosane and related compounds have been reported in the essential oil of Indian propolis [43]. Episesamin is a furofuran lignan that was reported by Bankova et al., in Canary Islands propolis [44]. o-Orsellinaldehyde, identified in Imathia sample, is a bioactive compound shown to exert cytotoxic effetcs against the human hepatoma hep B3 cells [45]. Consequently, the capacity of GC-MS to explore new constituents in natural products was verified in this work, unveiling eight new components of Greek propolis extracts.

### Antioxidant Activity, TPC, and TFC

The concentration of the extract that results in 50% of scavenging on DPPH was defined as IC_50_. The latter was obtained from the linear regression equation of equation constructed from the concentrations of the sample extracts and the inhibition percentage of radical scavenging activity. Lower IC_50_ values corroborate greater radical scavenging and antioxidant activity. The Imathia propolis extract displayed the lowest IC_50_ value of 1.19 μg/mL, hence the most prominent antioxidant activity. All IC_50_ values and TPC, TFC values are presented in Table 5.

**Table 5:** Antioxidant activity, total phenolic and total flavonoids content of propolis extracts.

Samples	Total Phenolic Content ^a^^,^^b^	Total Flavonoids Content ^a^^,^^c^	Scavenging Activity on DPPH IC_50_ (μg/mL)^a^	Normalized IC_50_ values (for PCA)^d^
Arkadia	127.8±11.2	52.2±1.9	8.91±0.14	13.36
Nafplio	155.5±9.4	73.3±2.1	2.66±0.51	44.74
Amorgos	113.2±7.1	59.0±3.8	14.90±2.09	7.99
Crete	110.2±5.4	63.6±0.7	16.11±3.18	7.39
Kos	159.2±13.8	57.3±3.3	2.43±0.61	48.97
Lakonia	120.3±4.4	46.8±1.5	11.7±2.39	10.17
Imathia	181.0±7.8	86.0±2.9	1.19±0.29	100.00
Kerkira	144.2±10.5	52.1±0.7	7.46±0.33	15.95
Quercetin	-	-	0.46±0.09	

^a^ Values represent the mean of triplicate measurements ± standard deviation

^b^ expressed as mg GAE/g_dry extract_

^c^ Results in mg quercetin/g_dry extract_

^d^ Normalization of IC_50_, setting value for Imathia at 100, calculating the rest accordingly.

### Principal Component Analysis

The main statistical procedure followed in this work, was principal component analysis (PCA). It was employed in order to investigate possible relationships or groupings between propolis locations and the analysed compounds.

**Fig 10:**
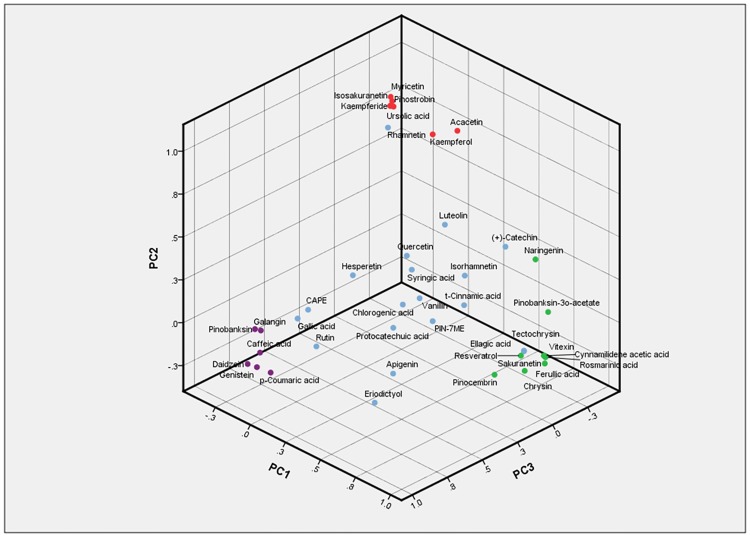
First three component plot in rotated space.

Two PCAs were applied and in both Varimax with Kaiser normalization method of rotation was selected. The first one involved the concentrations of all analytes as measured in the eight propolis locations. Seven principal components extracted by the analysis given that their eigenvalues exceed one (Kaiser’s rule). The first two components explained 50.8% of the total variance in initial values, while the following two accounted for 17.4% and 14.1% respectively (in total 82.3%). According to the rotated component matrix (“Table B in S1 File,” accompanied by the necessary correlation matrix “S3 File”) several analytes can be grouped having high (close to one) positive loadings in 1 to 7 principal components. In example, the first component is characterized by a high influence (loading>0.8) of Pinocembrin, Chrysin, Ferullic acid, Resveratrol, Naringenin, Pinobanksin-3o-acetate, Sakuranetin, Vitexin, Rosmarinic acid and Cynnamilidene acetic acid. Respectively, the second component is majorly driven by Pinostrobin, Kaempferol, Kaempferide, Acacetin, Isosakuranetin, Myricetin, and Ursolic acid while the third one is mainly related to Galangin, p-Coumaric acid, Pinobanksin, Caffeic acid, Daidzein and Genistein (Fig 10).

**Fig 11:**
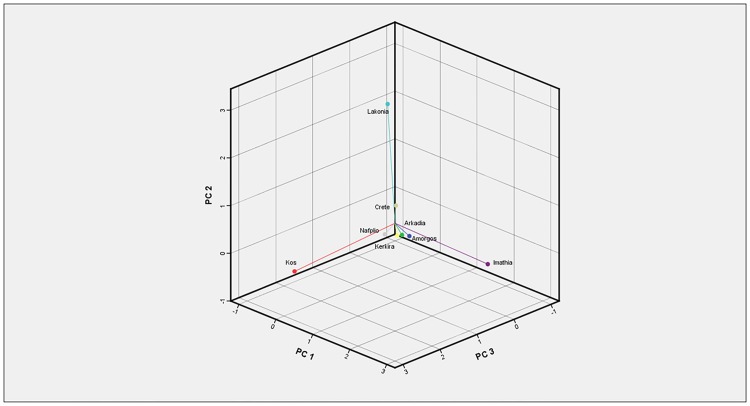
Plot of location’s scores in first three principal components.

The component scores of the propolis locations revealed no further grouping between sites, as each of them presented high values in distinct components. According to the analysis’ results, Imathia, Lakonia and Kos succeeded high scores in component 1, 2 and 3 respectively (Fig 11), which revealed an interrelation of those locations with the propolis compounds which demonstrate high influence on component 1, 2 and 3 as aforementioned.

**Fig 12:**
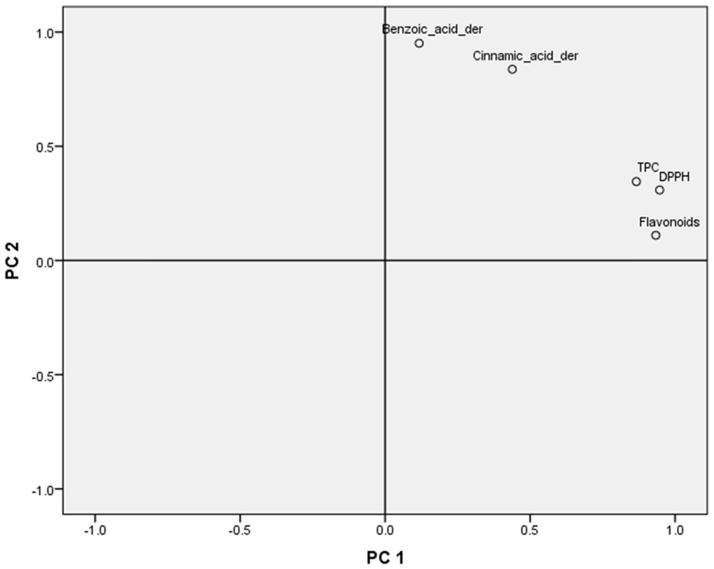
Component plot in rotated space.

**Fig 13:**
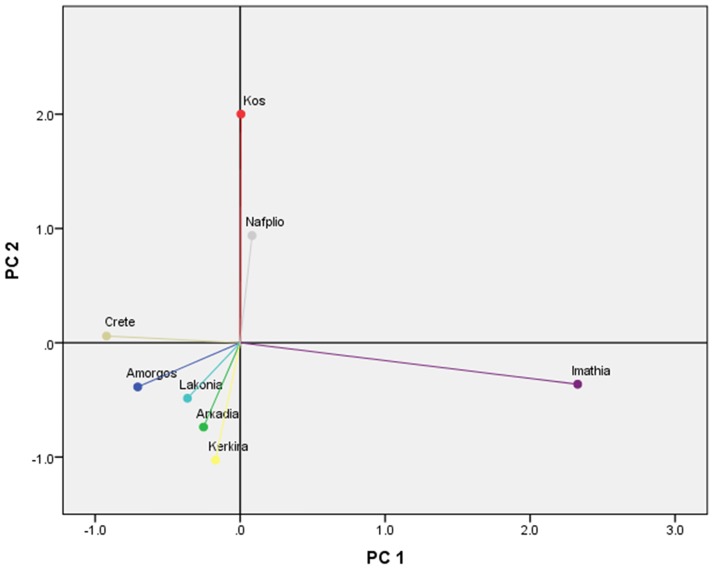
Plot of location’s scores in two principal components.

The second PCA regarded three classes of compounds, DPPH, TPC, and yet the eight propolis locations (see respective Figs 12 and 13). Three major classes of compounds, namely flavonoids, benzoic acid and cinnamic acid derivatives were selected. The selection was based on their occurrence in the studied propolis samples. For each class the sum of concentrations of individual components were calculated, for each propolis sample-location.

In this analysis, the principal components extracted, according to Kaiser’s rule which retains only factors with eigenvalues that exceed one, were two which cumulatively explained 91.1% of the variance in the initial data. Further, component loadings in the rotated space clearly revealed two groups (see Fig 12). The first one comprising of Flavonoids, DPPH, and TPC has high positive loadings in component 1 while the Cinnamic—Benzoic acid group most influences component 2 (the correlation matrix between variables, and component loadings after varimax rotation are presented in supplementary material “Tables C and D in S1 File” correspondingly).

This principal component analysis was consistent with the first one in highlighting Imathia and Kos as two locations having a unique behaviour. Indeed, Imathia performed a high score in component 1 demonstrating that Flavonoids, DPPH, and TPC are strongly related to its propolis compounds. On the other hand, Kos’s propolis is correlated with the Cinnamic—Benzoic acid group.

## Discussion

### Analytical Method and Results

The main aim of this work was the development of a novel analytical method for the identification and quantification of several phenolic compounds in raw propolis. Considering that these compounds are found in several plants and products, this method can serve as a universal tool in phytochemical analysis. For the identification and quantification of compounds, an appropriate extraction protocol should be applied with satisfactory extraction efficiency. Concerning extraction of compounds from raw propolis is known that several solvents are used in efforts to explore its chemical profile, and the different constituents or variations of them depending on the solvent or combinations of solvents used. When liquid chromatographic analysis is employed, the majority of propolis’ published works report ethanol or methanol or respective mixtures with water (where alcohol predominates) as the extraction solvent. Consequently, in the presented work an ethanol: water mixture (4:1) was selected for the extraction. Other solvents such as ethyl acetate, or dichloromethane were used in this work on a pilot basis, but did not show substantial differences in terms of quantities and number of compounds extracted, hence not further investigated.

The targeted analysis presented in this work has disclosed several new active substances, never reported in Greek propolis. Regions for which previous reports for propolis samples exist, and were also considered in this report, were Kos, Lakonia, Crete, Arkadia, and Kerkira. To evaluate and interpret results, it is worth to mention, that the sampling location (within a particular region) seems to detrimentally interplay in the chemical composition of samples. Therefore, variations between research works conducted within specific regions are expected to occur since it is unlike to refer in all studies, to the exact identical location. These variations are reflected by the possible discrepancies in terms of compounds detected, and respective concentrations. Another factor that should be regarded is that bees tend to select plants that are resin donors and possible year variations regarding such plants would impact their foraging preference. Last but not least, season in which propolis is collected by the bees is also a key determinant factor for its composition. Consequently, any differences observed, reflect to an extent, the differential foraging activity of bees within a specific region and period of time. Based on the above considerations, the incidence of the newly identified compounds in Greek regions also studied in the past are presented in Table 6.

**Table 6:** New compounds identified by HPLC-PDA-ESI/MS, in Greek regions (also investigated in the past).

Location	Kos*	Arkadia*^,^**	Lakonia*	Crete*^,^**	Kerkira***
Compounds	p-coumaric acid	PIN-7ME	CAPE	Apigenin	Kaempferol
	Caffeic acid	CAPE	Acacetin	Galangin	Rutin
	CAPE	Syringic acid	Myricetin	Isorhamnetin	Vanillin
	Apigenin	Pinostrobin	Pinostrobin	Rhamnetin	Rosmarinic acid
	Pinocembrin	Isorhamnetin	Kaempferide	CAPE	
	Eriodictyol	Eriodictyol		Luteolin	
	Kaempferol			Hesperetin	
	Galangin				
	Pinobanksin				
	Hesperetin				

* Graikou et al. 2016,

** Kalogeropoulos et al. 2009,

*** Çelemli et al. 2013

### Bioactivities of Constituents

In this paper, a brief overview of the bioactivities demonstrated by newly identified constituents of Greek propolis is provided. Hence, many of the new compounds, exhibit substantial activity as components of active extracts or as separately studied compounds. Indicatively, 1-octacosanol is a long-chain aliphatic alcohol that was stated to exhibit antio-angiogenic activity [48] and antioxidant activity [49]. With regard to azulene derivatives, extracts or natural products (such as chamomile tea) containing them (such as the one detected in Amorgos sample) are known for the significant bioactivity that they exhibit (indicatively see [50]).

(+)-Episesamin detected in Cretan sample, was found to exert antineoplastic effects in human hepatocellular carcinoma cell lines [51] and anti-inflammatory effects via inhibition of adipogenesis [52]. Eudesmic acid was found as a constituent of methanol extract of *Abutilon Indicum* leaves. The latter demonstrated moderate antibacterial activity [53]. Cinnamylidene acetic acid is usually used as a building block for the construction of bioactive compounds [54].

In this work, the first report of rosmarinic acid in Greek propolis is made (Kerkira sample). Rosmarinic acid is a constituent of several aromatic plants [55] and has also displayed antimutagenic activity as this was evaluated by the micronucleus assay in mice [56]. Rosemary extracts, rich in rosmarinic acid, also demonstrated antioxidant and antimicrobial properties [57]. Hesperetin is a plant bioflavonoid that is abundant in citrus fruits [58]. It exerts significant pharmacological properties, such as antioxidant and anti-inflammatory properties. Isorhamnetin a flavonol that was also detected exhibits significant bioactivity. More specifically, isorhamnetin was reported to show cytotoxic effects on human colon cancer cells [59]. Isosakuranetin is reported in propolis samples and in several plants belonging to divergent botanical families [60]. In addition, it was reported to have the potential to act as a protective agent for skin photoaging [61].

Other known constituents that were identified, are also bioactive and contribute to the bioactive profile of propolis. Indicatively, ferruginol (identified by GC-MS) is an abietane diterpene that along with several of its derivatives, in ethanolic extracts displayed cytotoxic activity against human tumor cells [62].

In this regard, first reports of several compounds in Greek propolis, highlight the chemical diversity of Greek propolis and postulate that revisiting natural products can lead to exploration of news constituents with pronounced biological activity, opening new frontiers to their exploitation.

### Biosynthetic Pathways of Newly Detected Compounds

In this section, a brief reference to biosynthetic pathways that might lead to the formation of several compounds identified in this work will be provided. Flavonoids (referred also as bioflavonoids), which is the major category of compounds determined in this work, are secondary metabolic products, therefore they have no straightforward implication with the development of plants. Flavonoids’ precursor molecule is phenylalanine. The latter is deaminated-transformed to cinnamic acid by the enzyme, phenylalanine ammonia lyase [63]. With regard to the newly identified flavonoids, acacetin is an aglycone that can be derived from hydrolysis of the respective flavonoid glycoside from the leaves of some plants such as *Robinia Pseudacacia* [64]. Kaempferide that is the 4’-O-methyl derivative of kaempferol was detected in one out of the eight samples implying that kaempferol, as a plant constituent, might have undergone the respective methylation. Kaempferide was reported as propolis constituent in Italian, Ukranian and FYROM propolis in a 2008 work [65], albeit is usually reported as a component of Brazilian propolis (indicatively see [35]). Sakuranetin is biosynthesized from naringenin, via the action of two agents, the S-adenosyl-L-methionine influenced by the enzyme naringenin-7-o-methyltransferase [66, 67]. Lately, sakuranetin was detected in Greek propolis (Graikou et al. 2016, in 3 regions Euboia, Evros, and Chalkidiki), but in this work, this compound is detected in a new studied region of Greece (Imathia). Isosakuranetin is also reported to be biosynthesized from naringenin *via* an *O*-methyltransferase enzyme expressed in E. coli [68]. Luteolin’s biosynthesis in *Rosmarinus* officinalis has been postulated by del Bano et al. [69]. In this work, naringenin was proposed to be hydroxylated to eriodictyol and finally converted to luteolin by the enzyme flavone synthase. Eriodictyol an aglyconic compound that was detected in Nafplio, Kos and Imathia propolis is a biotransformation product of naringenin, and hence, under specific conditions, it can be detected in propolis. Notwithstanding, naringenin was detected in all samples except Kos sample. Lin et al., reported pinostrobin (a flavanone glycoside), as a constituent of a 70% acetonic fraction of *Viscum angulatum* stems [70].

### Antioxidant Activity

Diphenylpicrylhydrazyl (DPPH) radical scavenging activity assay was implemented and assessed for all studied samples, exhibiting robust performance. Numerous groups have reported the use of the stable free radical DPPH for estimating antioxidant activity for several plant extracts and natural products. DPPH is considered as a stable free radical by virtue of the delocalization of the spare electron over the molecule as a whole so that the molecules do not form dimers, like most other free radicals. In the same context, the order of DPPH radical scavenging activity is in concordance with the total phenolic content, corroborating that antioxidant activity increased (lower IC_50_ values) with the increase of TPC.

With regard to the profound activity of Imathia propolis extract, the latter seems to be attributed to the high concentration of flavonoids that was portrayed both from targeted chemical analysis and TFC measurements. Imathia has one of the most fertile plains in Greece, listed along with Pella region, key areas of cultivation of peaches (an important crop for Greece with an export quantity of 155263 tones for 2012 based on FAO statistics, [71]). Nevertheless, peaches are bibliographically reported as a profound source of flavonoids [72] and as notable producers of both nectar and pollen that constitute them attractive to bees [73]. In the expected flight range of bees for the particular location that hives were positioned, several attractive crops, aromatic and edible wild plants are also abundant. In this context, apart from predominant peach trees, cotton trees, raspberries, chamomile, mentha, lavender, oregano are frequently found [74]. For Kos sample (the second most active propolis extract), it should be noted, that the sample originated from the North-East part of the Island that is the most prolific part with widespread cultivations of fruit trees (orange, mandarin, peach trees), and plants such as lavender, thyme, heather, sage and others that render this region an ideal area for foraging. On the other hand, although Crete is known for its diverse flora and abundance of fruit trees, the specific propolis extract displayed the lowest antioxidant activity. The latter is in line with the report of the Cretan beekeeper that had his hive positioned in a relatively isolated area targeting specific crops, a fact reflected in chemical analysis as well. Nonetheless, the explicit description of the flora of each studied region was not pursued since it was relatively difficult to monitor foraging bees and conclude to the most popular plants-flowers that they visited.

With regard to PCA, although it was not the primary aim of this work, it disclosed certain correlations that can be used as a basis for future studies. Such pursued works can underscore a more extensive sampling scheme within regions, and the addition of other antioxidant assays that will strengthen PCA with the inclusion of interrelated additional variables. The latter may unveil other correlations among components and the flora of the studied areas.

## Conclusions

The developed analytical method can serve as a universal tool to detect and identify common phenolic compounds that are not only encountered in propolis but also in other plants and their extracts. Hence, it has broad applicability and can be implemented in several projects that aim to elucidate chemical composition of beehive products, and other natural products and their extracts. A further step to improve this work, will be the adaptation of this approach to a high-resolution mass spectrometry scheme circumventing the issue of analytical standards availability and proceed to fingerprinting analysis *via* untagreted analysis of propolis samples elucidating all present compounds. Concerning the antioxidant activity of propolis extracts, a pronounced activity was evidenced for some of the extracts, with two of them displaying IC_50_ values below 2.5 μM, comparably active to that of quercetin. The demonstrated activity along with the unveiling of new substances present the necessity to explore propolis and other apiculture products continuously. Last but not least, targeted bioassays are currently underway, to further explore the bioactivity of these extracts.

## Supporting Information

S1 File “Figures A-F”, and “Tables A-D”. (DOCX)

S2 File “Figures A-Z, A1-Z1 and A2-C2”. (DOCX)

S3 File Correlation Matrix between compounds. (DOCX)
