# Supplementary material for: Revisiting Greek Propolis: Chromatographic Analysis and Antioxidant Activity Study
Source: PLoS One. 2017 Jan 19;12(1):e0170077. doi: 10.1371/journal.pone.0170077 (PMC5245904; doi:10.1371/journal.pone.0170077)
Supplement: S1 File — (DOCX) [file pone.0170077.s001.docx]

**Supplementary material**

**Fig A.** Pinostrobin chalcone and pinocembrin GC-MS mass spectra

| Pinostrobin chalcone |  |
| --- | --- |
|  |  |
| pinocembrin |  |
|  |  |

**Fig B.** Eudesmic acid, azulenone derivative, o-Orsellinaldehyde and (+)-episesamin GC-MS mass spectra

| Eudesmic acid | |  |
| --- | --- | --- |
|  | |  |
| 3,8-Dimethyl-4-(1-methylethylidene)-2,4,6,7,8,8a-hexahydro-5(1H)-azulenone |  | |
|  |  | |
| o-Orsellinaldehyde |  | |
|  |  | |
| (+)-Episesamin |  | |
|  |  | |

**Fig C.** HPLC-UV chromatogram of Nafplio propolis extract at 335 nm.

**Fig D.** SIM chromatograms of Crete propolis extract

**Fig E**. (a) Full Scan ESI(+) MS chromatogram of Brazilian Propolis Tincture containing Artepillin C


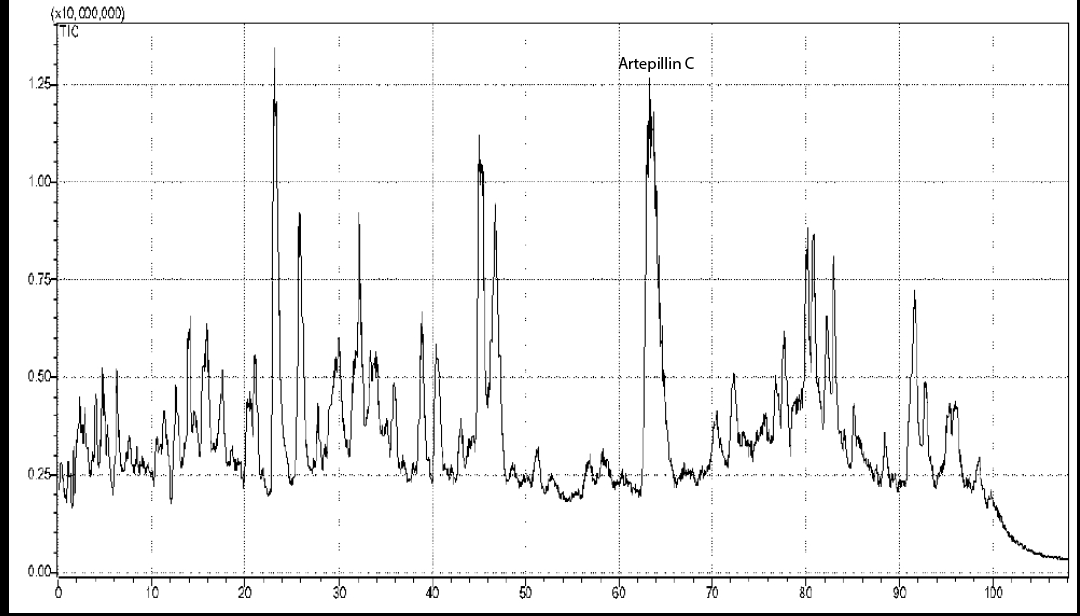


**Fig F.** Selected SIM chromatograms of techtochrysin, corosolic acid, maslinic acid, and betulinic acid in standard solution at 100 ng/mL.

**Table A.** Tentative characterization of compounds from Full Scan MS

| Basic Fragment Ion | Time (min) | Other fragment ions | Compound |
| --- | --- | --- | --- |
| 285 | 12.27 | - | Pinobanksin 5-methyl ether |
| 315 | 13.26 | 301, 271 | Quercetin3-methyl ether |
| 267 | 14.59 | 253, 223 | Chrysin-5-methyl ether |
| 299 | 18.05 | 301, 300, 284, 271 | Kaempferol methyl ether |
| 329 | 19.02 | 314, 311, 299, 271, 249, 243 | Quercetin dimethyl ether |
| 283 | 20.51 | 284, 243, 239, 211 | Galangin 5-methyl-ether |
| 327 | 21.21 | 285, 267 | Pinobanksin 5-methyl ether-3-O-acetate |
| 329 | 25.45 | 314, 249, 243 | Quercetin dimethyl ether |
| 269 | 29.77 | 255, 247, 227 | Pinocembrin 5-methyl ether |
| 295 | 42.77 | 178, 134 | Caffeic acid cinnamyl ester |
| 327 | 45.76 | 271, 253 | Pinobanksin 3-*O*-propionate |
| 341 | 61.80 | 253 | Pinobanksin 3-*O*-butyrate or isobutyrate |
| 353 | 64.84 | 253 | Pinobanksin 3-*O*-pentenoate |

| **Table B.** Rotated Component Matrix^a^ | | | | | | | |
| --- | --- | --- | --- | --- | --- | --- | --- |
|  | Component | | | | | | |
|  | 1 | 2 | 3 | 4 | 5 | 6 | 7 |
| Pinocembrin | .872 | -.107 | .209 | .331 | .247 | .053 | -.105 |
| Apigenin | .669 | .029 | .729 | -.091 | .104 | .012 | -.018 |
| Chrysin | .915 | -.137 | .037 | .263 | -.161 | .182 | -.121 |
| Galangin | -.097 | .040 | .906 | .393 | -.044 | -.100 | .036 |
| Ellagic acid | .701 | -.195 | -.171 | -.214 | -.116 | -.329 | -.524 |
| Tectochrysin | .745 | -.156 | -.128 | -.132 | -.194 | .590 | -.024 |
| Syringic acid | -.210 | -.137 | -.283 | -.142 | .330 | .851 | -.061 |
| Ferullic acid | .978 | -.101 | -.043 | -.066 | -.009 | -.008 | -.166 |
| Gallic acid | -.229 | -.105 | .512 | .788 | -.173 | -.019 | .155 |
| Hesperetin | -.259 | -.038 | .088 | -.002 | .941 | .153 | -.120 |
| Luteolin | .308 | .452 | -.001 | .796 | -.231 | -.115 | .036 |
| p-Coumaric acid | -.107 | -.242 | .826 | .485 | -.100 | .024 | .051 |
| Pinobanksin | -.069 | .088 | .976 | -.057 | .178 | .019 | .017 |
| PIN-7ME | .046 | -.289 | -.176 | .851 | -.332 | .029 | .218 |
| Caffeic acid | -.041 | -.041 | .968 | -.173 | .131 | -.056 | .096 |
| Pinostrobin | -.074 | .978 | -.017 | -.057 | -.180 | -.042 | .031 |
| CAPE | -.318 | -.157 | .348 | -.284 | .205 | -.280 | .743 |
| Quercetin | -.260 | -.082 | -.298 | .445 | -.229 | -.353 | .680 |
| Rhamnetin | -.194 | .774 | -.097 | .326 | -.420 | .264 | .043 |
| Kaempferol | .210 | .934 | -.012 | -.040 | .263 | -.050 | -.100 |
| Chlorogenic acid | -.069 | -.199 | -.078 | .956 | .175 | -.065 | -.003 |
| Protocatechuic acid | .408 | .083 | .467 | -.130 | .765 | -.041 | .064 |
| Kaempferide | -.092 | .977 | -.012 | -.052 | -.171 | -.064 | .031 |
| Acacetin | .167 | .848 | -.231 | -.085 | .412 | -.081 | .126 |
| Resveratrol | .804 | -.128 | -.047 | .563 | -.066 | -.099 | -.057 |
| Eriodictyol | .588 | -.153 | .779 | -.106 | .103 | -.042 | -.010 |
| Naringenin | .820 | .404 | -.135 | .293 | -.220 | .007 | .107 |
| Pinobanksin-3o-acetate | .838 | .075 | -.207 | -.179 | -.048 | -.197 | .419 |
| (+)-Catechin | .727 | .489 | -.014 | -.144 | -.235 | .396 | -.009 |
| Rutin | -.182 | -.284 | .425 | -.325 | -.317 | .704 | -.060 |
| Isorhamnetin | .298 | .089 | -.153 | .919 | .153 | -.064 | -.083 |
| Sakuranetin | .993 | -.041 | -.023 | .018 | .074 | -.054 | -.062 |
| Isosakuranetin | -.200 | .911 | -.133 | -.026 | .331 | .029 | -.042 |
| Daidzein | -.130 | -.144 | .967 | -.136 | .075 | -.037 | .039 |
| Vitexin | .993 | -.041 | -.023 | .018 | .074 | -.054 | -.062 |
| Rosmarinic acid | .985 | -.061 | -.041 | -.009 | .053 | -.088 | -.119 |
| Myricetin | -.146 | .984 | -.069 | -.042 | .059 | -.023 | -.002 |
| Ursolic acid | -.092 | .977 | -.012 | -.052 | -.171 | -.064 | .031 |
| Genistein | -.158 | -.212 | .873 | -.217 | -.076 | .335 | .053 |
| Cynnamilidene acetic acid | .993 | -.041 | -.023 | .018 | .074 | -.054 | -.062 |
| t-Cinnamic acid | .106 | -.238 | -.340 | -.293 | .246 | -.323 | -.752 |
| Vanillin | -.250 | -.359 | -.379 | -.470 | -.258 | -.613 | -.039 |
| Extraction Method: Principal Component Analysis.  Rotation Method: Varimax with Kaiser Normalization. | | | | | | | |
| 1. Rotation converged in 6 iterations. | | | | | | | |

**Table C.** Correlation matrix between variables.

| **Correlation Matrix** | | | | | |
| --- | --- | --- | --- | --- | --- |
|  | Flavonoids | Cinnamic_acid_der | Benzoic_acid_der | DPPH | TPC |
| Flavonoids | 1.000 | 0.469 | 0.272 | 0.915 | 0.733 |
| Cinnamic_acid_der | 0.469 | 1.000 | 0.759 | 0.666 | 0.671 |
| Benzoic_acid_der | 0.272 | 0.759 | 1.000 | 0.410 | 0.396 |
| DPPH | 0.915 | 0.666 | 0.410 | 1.000 | 0.924 |
| TPC | 0.733 | 0.671 | 0.396 | 0.924 | 1.000 |

**Table D.** Component loadings after varimax rotation was applied.

|  | Component | |
| --- | --- | --- |
|  | 1 | 2 |
| Flavonoids | 0.933 | 0.110 |
| Cinnamic_acid_der | 0.439 | 0.837 |
| Benzoic_acid_der | 0.118 | 0.951 |
| DPPH | 0.947 | 0.309 |
| TPC | 0.866 | 0.346 |
