# Supplementary material for: Revisiting Greek Propolis: Chromatographic Analysis and Antioxidant Activity Study
Source: PLoS One. 2017 Jan 19;12(1):e0170077. doi: 10.1371/journal.pone.0170077 (PMC5245904; doi:10.1371/journal.pone.0170077)
Supplement: S2 File — (DOCX) [file pone.0170077.s002.docx]

**SIM Chromatograms with m/z values**

Fig A. Protocatechuic acid

Fig B. Pinocembrin

Fig C. Kaempferol

Fig D. Apigenin

Fig E. Chrysin

Fig F. Galangin

Fig G. Chlorogenic acid

Fig H. Daidzein

Fig I. Ellagic acid

Fig J. Ferrulic acid

Fig K. Gallic acid

Fig L. Hesperetin

Fig M. Hydroxytyrosol

Fig N. Luteolin

Fig O. p-Coumaric acid

Fig P. Pinobanksin

Fig Q. Pin-7-ME

Fig R. Quercetin

Fig S. Techtochrysin

Fig T. Caffeic acid

Fig U. Sakuranetin

Fig V. Rhamnetin

Fig W. Pinostrobin

Fig X. Syringic acid

Fig Y. Kaempferide

Fig Z. Acacetin

Fig A1. Rutin

Fig B1. Protocatechuic acid ethyl ester

Fig C1. Resveratrol

Fig D1. Phloridzin

Fig E1. Naringenin

Fig F1. Eriodictyol

Fig G1. Diosmetin

Fig H1. Rosmarinic acid

Fig I1. Myricetin

Fig J1. Isorhamnetin

Fig K1. Isosakuranetin

Fig L1. Catechin (in modified prolonged method)

Fig M1. Orientin


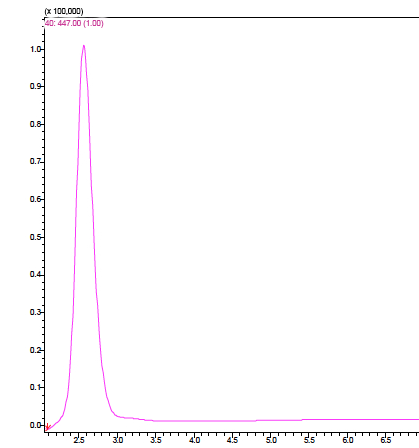


Fig N1. Vitexin

Fig O1. t-Cinnamic acid

Fig P1. Pinobanksin 3o-acetate

Fig Q1. Cinnamilidene acetic acid


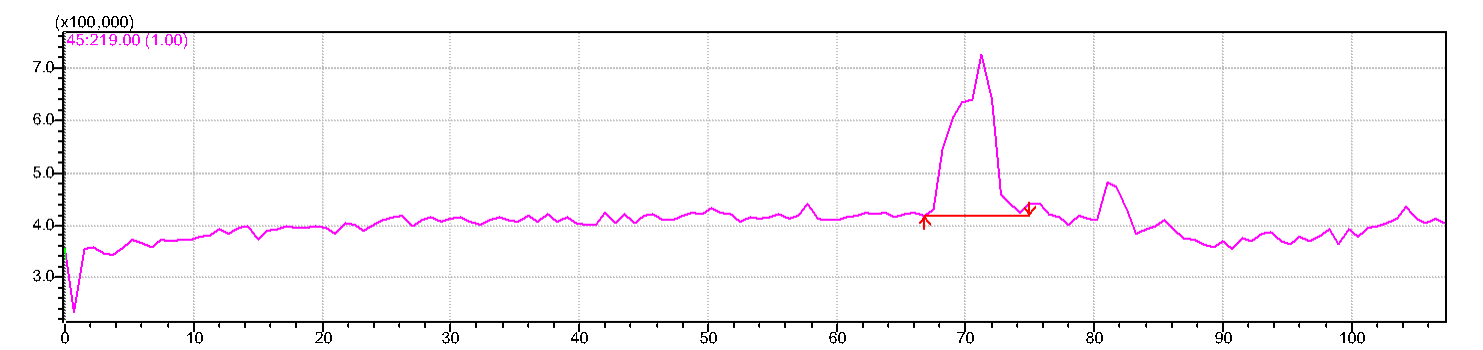


Fig R1. Artepillin c

Fig S1. Adipic acid

Fig T1. Ursolic acid

Fig U1. Suberic acid

Fig V1. Genistein

Fig W1. Hesperidin

Fig X1. Isoferulic acid

Fig Y1. Tangeretin

Fig Z1. Diosmin

Fig A2. Vanillin

Fig B2. Chrysoeriol

Fig C2. Naringin
