# Supplementary material for: Revisiting Greek Propolis: Chromatographic Analysis and Antioxidant Activity Study
Source: PLoS One. 2017 Jan 19;12(1):e0170077. doi: 10.1371/journal.pone.0170077 (PMC5245904; doi:10.1371/journal.pone.0170077)
Supplement: S3 File — (DOCX) [file pone.0170077.s003.docx]

**S1 Table. Correlation Matrix between compounds**

|  | Pinocembrin | Apigenin | Chrysin | Galangin |  |
| --- | --- | --- | --- | --- | --- |
| Correlation Pinocembrin Apigenin Chrysin Galangin Ellagic acid Τectochrysin Syringic acid Ferullic acid Gallic acid Hesperetin Luteolin  p-Coumaric acid  Pinobanksin PIN-7ME Caffeic acid Pinostrobin CAPE Quercetin Rhamnetin Kaempferol  Chlorogenic acid  Protocatechuic acid  Kaempferide | 1.000  .731  .890  .210  .535  .583  -.142  .848  .119  .049  .417  .237  .158  .213  .133  -.241  -.324  -.279  -.258  .140  .301  .582  -.253 | .731  1.000  .598  .555  .352  .400  -.292  .628  .124  -.008  .120  .469  .692  -.222  .705  -.048  .067  -.474  -.249  .191  -.178  .707  -.056 | .890  .598  1.000  .027  .628  .806  -.112  .910  .036  -.338  .441  .108  -.082  .331  -.084  -.200  -.505  -.230  -.091  .013  .173  .207  -. 220 | .210  .555  .027  1.000  -.295  -.298  -.399  -.168  .806  .041  .322  .943  .863  .179  .815  .022  .275  -.003  .084  -.020  .303  .309  .031 |  |

|  | Ellagic acid | τectochrysin | Syringic acid | Ferullic acid |
| --- | --- | --- | --- | --- |
| Correlation Pinocembrin Apigenin Chrysin Galangin Ellagic acid Tectochrysin Syringic acid Ferullic acid Gallic acid Hesperetin Luteolin  p-Coumaric acid  Pinobanksin PIN-7ME Caffeic acid Pinostrobin CAPE Quercetin Rhamnetin Kaempferol  Chlorogenic acid  Protocatechuic acid  Kaempferide | .535  .352  .628  -.295  1.000  .444  -.328  .817  -.451  -.286  .004  -.295  -.256  -.149  -.196  -.208  -.512  -.424  -.400  .014  -.198  .109  -.216 | .583  .400  .806  -.298  .444  1.000  .360  .760  -.305  -.287  .030  -.179  -.206  .065  -.186  -.189  -.443  -.382  -.060  -.061  -.209  .075  -.218 | -.142  -.292  -.112  -.399  -.328  .360  1.000  -.170  -.276  .482  -.416  -.263  -.191  -.139  -.246  -.203  -.185  -.331  -.001  -.113  -.070  .002  -.217 | .848  .628  .910  -.168  .817  .760  -.170  1.000  -.311  -.242  .201  -.155  -.119  -.008  -.083  -.170  -.414  -.370  -.291  .129  -.108  .362  -.187 |

|  | Gallic acid | Hesperetin | Luteolin | **p-Coumaήc acid** |
| --- | --- | --- | --- | --- |
| Correlation Pinocembrin Apigenin Chrysin Galangin Ellagic acid Tectochrysin Syringic acid Ferullic acid Gallic acid Hesperetin Luteolin  p-Coumaric acid  Pinobanksin PIN-7ME Caffeic acid Pinostrobin CAPE Quercetin Rhamnetin Kaempferol  Chlorogenic acid  Protocatechuic acid  Kaempferide | .119  .124  .036  .806  -.451  -.305  -.276  -.311  1.000  -.077  .555  .879  .432  .691  .366  -.103  .129  .418  .245  -.244  .721  -.087  -.093 | .049  -.008  -.338  .041  -.286  -.287  .482  -.242  -.077  1.000  -.338  .012  .269  -.352  .201  -.198  .181  -.308  -.348  .161  .172  .639  -.188 | .417  .120  .441  .322  .004  .030  -.416  .201  .555  -.338  1.000  .265  -.071  .642  -.190  .421  -.383  .355  .617  .396  .617  -.11ο  .420 | .237  .469  .108  .943  -.295  -.179  -.263  -.155  .879  .012  .265  1.000  .747  .378  .720  -.252  .232  .067  -.038  -.310  .436  .185  -.245 |

|  | Pinobanksin | PIN-7ME | Caffeic acid | Pinostrobin |
| --- | --- | --- | --- | --- |
| Correlation Pinocembrin Apigenin Chrysin Galangin Ellagic acid Tectochrysin Syringic acid Ferullic acid Gallic acid Hesperetin Luteolin  p-Coumaric acid  Pinobanksin PIN-7ME Caffeic acid Pinostrobin CAPE Quercetin Rhamnetin Kaempferol  Chlorogenic acid  Protocatechuic acid  Kaempferide | .158  .692  -.082  .863  -.256  -.206  -.191  -.119  .432  .269  -.071  .747  1.000  -.304  .977  .046  .408  -.341  -.101  .103  -.114  .579  .053 | .213  -.222  .331  .179  -.149  .065  -.139  -.008  .691  -.352  .642  .378  -.304  1.000  -.332  -.267  -.186  .657  .218  -.403  .822  -.439  -.267 | .133  .705  -.084  .815  -.196  -.186  -.246  -.083  .366  .201  -.190  .720  .977  -.332  1.000  -.061  .519  -.296  -.240  -.024  -.203  .563  -.055 | -.241  -.048  -.200  .022  -.208  -.189  -.203  -.170  -.103  -.198  .421  -.252  .046  -.267  -.061  1.000  -.122  -.005  .820  .852  -.272  -.083  1.000 |

|  | CAPE | Quercetin | Rhamnetin | Kaempferol |
| --- | --- | --- | --- | --- |
| Correlation Pinocembrin Apigenin Chrysin Galangin Ellagic acid Tectochrysin Syringic acid Ferullic acid Gallic acid Hesperetin Luteolin  p-Coumaric acid  Pinobanksin PIN-7ME Caffeic acid Pinostrobin CAPE Quercetin Rhamnetin Kaempferol  Chlorogenic acid  Protocatechuic acid  Kaempferide | -.324  .067  -.505  .275  -.512  -.443  -.185  -.414  .129  .181  -.383  .232  .408  -.186  .519  -.122  1.000  .422  -.314  -.213  -.193  .273  -.108 | -.279  -.474  -.230  -.003  -.424  -.382  -.331  -.370  .418  -.308  .355  .067  -.341  .657  -.296  -.005  .422  1.000  .193  -.256  .464  -.427  .006 | -.258  -.249  -.091  .084  -.400  -.060  -.001  -.291  .245  -.348  .617  -.038  -.101  .218  -.240  .820  -.314  .193  1.000  .543  .088  -.432  .814 | .140  .191  .013  -.020  .014  -.061  -.113  .129  -.244  .161  .396  -.31ο  .103  -.403  -.024  .852  -.213  -.256  .543  1.000  -.189  .360  .851 |

|  | **Chlorogenic**  **acid** | **Protocatechui**  **cacid** | Kaempferide | Acacetin |  |
| --- | --- | --- | --- | --- | --- |
| Correlation Pinocembrin Apigenin Chrysin Galangin Ellagic acid Tectochrysin Syringic acid Ferullic acid Gallic acid Hesperetin Luteolin  p-Coumaric acid  Pinobanksin PIN-7ME Caffeic acid Pinostrobin CAPE Quercetin Rhamnetin Kaempferol  Chlorogenic acid  Protocatechuic acid  Kaempferide | .301  -.178  .173  .303  -.198  -.209  -.070  -.108  .721  .172  .617  .436  -.114  .822  -.203  -.272  -.193  .464  .088  -.189  1.000  -.069  -.263 | .582  .707  .207  .309  .109  .075  .002  .362  -.087  .639  -.11ο  .185  .579  -.439  .563  -.083  .273  -.427  -.432  .360  -.069  1.000  -.081 | -.253  -.056  -.220  .031  -.216  -.218  -.217  -.187  -.093  -.188  .420  -.245  .053  -.267  -.055  1.000  -.108  .006  .814  .851  -.263  -.081  1.000 | .063  .016  -.091  -.230  -.078  -.098  -.015  .069  -.363  .265  .286  -.491  -.083  -.381  -.179  .759  -.042  -.063  .430  .934  -.167  .368  .759 |  |

|  | Resveratrol | Eriodictyol | Naringenin | **Pinobanksin-**  **3o-acetate** |
| --- | --- | --- | --- | --- |
| Correlation Pinocembrin Apigenin Chrysin Galangin Ellagic acid Tectochrysin Syringic acid Ferullic acid Gallic acid Hesperetin Luteolin  p-Coumaric acid  Pinobanksin PIN-7ME Caffeic acid Pinostrobin CAPE Quercetin Rhamnetin Kaempferol  Chlorogenic acid  Protocatechuic acid  Kaempferide | .876  .442  .899  .106  .547  .507  -.321  .775  .253  -.279  .663  .181  -.159  .568  -.179  -.202  -.440  .078  -.068  .020  .507  .173  -.212 | .681  .977  .537  .601  .338  .332  -.309  .565  .178  .014  .008  .555  .730  -.193  .770  -.217  .164  -.434  -.398  .005  -.151  .685  -.223 | .676  .411  .791  -.058  .424  .571  -.305  .732  -.014  -.459  .723  -.127  -.207  .290  -.250  .362  -.422  .045  .361  .471  .114  .106  .347 | .554  .414  .623  -.299  .498  .546  -.312  .765  -.370  -.363  .199  -.344  -.248  .002  -.161  .055  .057  .123  -.157  .211  -.225  .274  .042 |

|  | (+) -Catechin | Rutin | lsorhamnetin | Sakuranetin |
| --- | --- | --- | --- | --- |
| Correlation Pinocembrin Apigenin Chrysin Galangin Ellagic acid Tectochrysin Syringic acid Ferullic acid Gallic acid Hesperetin Luteolin  p-Coumaric acid  Pinobanksin PIN-7ME Caffeic acid Pinostrobin CAPE Quercetin Rhamnetin Kaempferol  Chlorogenic acid  Protocatechuic acid  Kaempferide | .495  .483  .670  -.150  .350  .765  .065  .672  -.307  -.367  .338  -.245  -.047  -.141  -.092  .458  -.438  -.381  .394  .535  -.352  .153  .433 | -.182  .186  -.011  .205  -.238  .376  .502  -.139  .065  -.088  -.454  .326  .377  -.166  .400  -.227  .036  -.418  -.016  -.408  -.376  -.132  -.242 | .566  .023  .470  .193  .068  .042  -.161  .242  .530  .048  .833  .245  -.190  .727  -.307  -.012  -.435  .301  .241  .162  .884  .053  -.011 | .893  .653  .903  -.111  .745  .702  -.217  .984  -.242  -.188  .289  -.117  -.084  .037  -.057  -.127  -.338  -.280  -.264  .198  -.025  .444  -.143 |

|  | **lsosakuraneti**  **η** | Daidzein | Vitexin | **Rosmarinic**  **acid** |  |
| --- | --- | --- | --- | --- | --- |
| Correlation Pinocembrin Apigenin Chrysin Galangin Ellagic acid Tectochrysin Syringic acid Ferullic acid Gallic acid Hesperetin Luteolin  p-Coumaric acid  Pinobanksin PIN-7ME Caffeic acid Pinostrobin CAPE Quercetin Rhamnetin Kaempferol  Chlorogenic acid  Protocatechuic acid  Kaempferide | -.221  -.166  -.363  -.094  -.315  -.317  .095  -.277  -.202  .327  .248  -.355  .025  -.389  -.115  .847  -.090  -.11ο  .615  .901  -.126  .185  .851 | .071  .633  -.123  .832  -.216  -.218  -.217  -.152  .427  .184  -.226  .775  .961  -.267  .987  -.150  .495  -.280  -.264  -.151  -.152  .465  -.143 | .893  .653  .903  -.111  .745  .702  -.217  .984  -.242  -.188  .289  -.117  -.084  .037  -.057  -.127  -.338  -.280  -.264  .198  -.025  .444  -.143 | .875  .634  .895  -.137  .796  .690  -.236  .991  -.274  -.205  .263  -.142  -.107  .016  -.076  -.141  -.368  -.305  -.288  .181  -.046  .416  -.156 |  |

|  | Myricetin | Ursolic acid | Genistein | **Cynnamiliden e acetic acid** |
| --- | --- | --- | --- | --- |
| Correlation Pinocembrin Apigenin Chrysin Galangin Ellagic acid Tectochrysin Syringic acid Ferullic acid Gallic acid Hesperetin Luteolin  p-Coumaric acid  Pinobanksin PIN-7ME Caffeic acid Pinostrobin CAPE Quercetin Rhamnetin Kaempferol  Chlorogenic acid  Protocatechuic acid  Kaempferide | -.248  -.11ο  -.296  -.026  -.271  -.273  -.079  -.237  -.148  .047  .355  -.306  .042  -.335  -.085  .967  -.104  -.048  .752  .908  -.209  .041  .969 | -.253  -.056  -.220  .031  -.216  -.218  -.217  -.187  -.093  -.188  .420  -.245  .053  -.267  -.055  1.000  -.108  .006  .814  .851  -.263  -.081  1.000 | -.011  .540  -.073  .684  -.301  .044  .103  -.167  .349  .099  -.338  .701  .850  -.238  .874  -.196  .379  -.363  -.167  -.275  -.258  .286  -.199 | .893  .653  .903  -.111  .745  .702  -.217  .984  -.242  -.188  .289  -.117  -.084  .037  -.057  -.127  -.338  -.280  -.264  .198  -.025  .444  -.143 |

|  | **t-Cinnamic acid** | Vanillin |
| --- | --- | --- |
| Correlation Pinocembrin Apigenin Chrysin Galangin Ellagic acid Tectochrysin Syringic acid Ferullic acid Gallic acid Hesperetin Luteolin  p-Coumaric acid  Pinobanksin PIN-7ME Caffeic acid Pinostrobin CAPE Quercetin Rhamnetin Kaempferol  Chlorogenic acid  Protocatechuic acid  Kaempferide | .073  -.122  .032  -.449  .713  -.021  .001  .287  -.557  .225  -.355  -.447  -.318  -.372  -.294  -.272  -.449  -.491  -.488  -.028  -.148  .056  -.267 | -.506  -.445  -.382  -.447  .312  -.331  -.329  -.147  -.419  -.286  -.483  -.419  -.416  -.181  -.265  -.228  .226  .248  -.401  -.398  -.336  -.423  -.217 |

|  | Pinocembrin | Apigenin | Chrysin | Galangin |  |
| --- | --- | --- | --- | --- | --- |
| Acacetin Resνeratrol Eriodictyol Naringenin  Pinobanksin-3o-acetate  (+)-Catechin Rutin lsorhamnetin Sakuranetin lsosakuranetin Daidzein Vitexin  Rosmarinic acid  Myricetin Ursolic acid Genistein  **Cynnamilidene acetic acid**  t-Cinnamic acid  Vanillin | .063  .876  .681  .676  .554  .495  -.182  .566  .893  -.221  .071  .893  .875  -.248  -.253  -.011  .893  .073  -.506 | .016  .442  .977  .411  .414  .483  .186  .023  .653  -.166  .633  .653  .634  -.11ο  -.056  .540  .653  -.122  -.445 | -.091  .899  .537  .791  .623  .670  -.011  .470  .903  -.363  -.123  .903  .895  -.296  -.220  -.073  .903  .032  -.382 | -.230  .106  .601  -.058  -.299  -.150  .205  .193  -.111  -.094  .832  -.111  -.137  -.026  .031  .684  -.111  -.449  -.447 |  |

|  | Ellagic acid | τectochrysin | Syringic acid | Ferullic acid |
| --- | --- | --- | --- | --- |
| Acacetin Resνeratrol Eriodictyol Naringenin  Pinobanksin-3o-acetate  (+)-Catechin Rutin lsorhamnetin Sakuranetin lsosakuranetin Daidzein Vitexin  Rosmarinic acid  Myricetin Ursolic acid Genistein  **Cynnamilidene acetic acid**  t-Cinnamic acid  Vanillin | -.078  .547  .338  .424  .498  .350  -.238  .068  .745  -.315  -.216  .745  .796  -.271  -.216  -.301  .745  .713  .312 | -.098  .507  .332  .571  .546  .765  .376  .042  .702  -.317  -.218  .702  .690  -.273  -.218  .044  .702  -.021  -.331 | -.015  -.321  -.309  -.305  -.312  .065  .502  -.161  -.217  .095  -.217  -.217  -.236  -.079  -.217  .103  -.217  .001  -.329 | .069  .775  .565  .732  .765  .672  -.139  .242  .984  -.277  -.152  .984  .991  -.237  -.187  -.167  .984  .287  -.147 |

|  | Gallic acid | Hesperetin | Luteolin | **p-Coumaήc acid** |
| --- | --- | --- | --- | --- |
| Acacetin Resνeratrol Eriodictyol Naringenin  Pinobanksin-3o-acetate  (+)-Catechin Rutin lsorhamnetin Sakuranetin lsosakuranetin Daidzein Vitexin  Rosmarinic acid  Myricetin Ursolic acid Genistein  **Cynnamilidene acetic acid**  t-Cinnamic acid  Vanillin | -.363  .253  .178  -.014  -.370  -.307  .065  .530  -.242  -.202  .427  -.242  -.274  -.148  -.093  .349  -.242  -.557  -.419 | .265  -.279  .014  -.459  -.363  -.367  -.088  .048  -.188  .327  .184  -.188  -.205  .047  -.188  .099  -.188  .225  -.286 | .286  .663  .008  .723  .199  .338  -.454  .833  .289  .248  -.226  .289  .263  .355  .420  -.338  .289  -.355  -.483 | -.491  .181  .555  -.127  -.344  -.245  .326  .245  -.117  -.355  .775  -.117  -.142  -.306  -.245  .701  -.117  -.447  -.419 |

|  | Pinobanksin | PIN-7ME | Caffeic acid | Pinostrobin |
| --- | --- | --- | --- | --- |
| Acacetin Resνeratrol Eriodictyol Naringenin  Pinobanksin-3o-acetate  (+)-Catechin Rutin lsorhamnetin Sakuranetin lsosakuranetin Daidzein Vitexin  Rosmarinic acid  Myricetin Ursolic acid Genistein  **Cynnamilidene acetic acid**  t-Cinnamic acid  Vanillin | -.083  -.159  .730  -.207  -.248  -.047  .377  -.190  -.084  .025  .961  -.084  -.107  .042  .053  .850  -.084  -.318  -.416 | -.381  .568  -.193  .290  .002  -.141  -.166  .727  .037  -.389  -.267  .037  .016  -.335  -.267  -.238  .037  -.372  -.181 | -.179  -.179  .770  -.250  -.161  -.092  .400  -.307  -.057  -.115  .987  -.057  -.076  -.085  -.055  .874  -.057  -.294  -.265 | .759  -.202  -.217  .362  .055  .458  -.227  -.012  -.127  .847  -.150  -.127  -.141  .967  1.000  -.196  -.127  -.272  -.228 |

|  | CAPE | Quercetin | Rhamnetin | Kaempferol |
| --- | --- | --- | --- | --- |
| Acacetin Resνeratrol Eriodictyol Naringenin  Pinobanksin-3o-acetate  (+)-Catechin Rutin lsorhamnetin Sakuranetin lsosakuranetin Daidzein Vitexin  Rosmarinic acid  Myricetin Ursolic acid Genistein  **Cynnamilidene acetic acid**  t-Cinnamic acid  Vanillin | -.042  -.440  .164  -.422  .057  -.438  .036  -.435  -.338  -.090  .495  -.338  -.368  -.104  -.108  .379  -.338  -.449  .226 | -.063  .078  -.434  .045  .123  -.381  -.418  .301  -.280  -.11ο  -.280  -.280  -.305  -.048  .006  -.363  -.280  -.491  .248 | .430  -.068  -.398  .361  -.157  .394  -.016  .241  -.264  .615  -.264  -.264  -.288  .752  .814  -.167  -.264  -.488  -.401 | .934  .020  .005  .471  .211  .535  -.408  .162  .198  .901  -.151  .198  .181  .908  .851  -.275  .198  -.028  -.398 |

|  | **Chlorogenic acid** | **Protocatechui cacid** | Kaempferide | Acacetin |  |
| --- | --- | --- | --- | --- | --- |
| Acacetin Resνeratrol Eriodictyol Naringenin  Pinobanksin-3o-acetate  (+)-Catechin Rutin lsorhamnetin Sakuranetin lsosakuranetin Daidzein Vitexin  Rosmarinic acid  Myricetin Ursolic acid Genistein  **Cynnamilidene acetic acid**  t-Cinnamic acid  Vanillin | -.167  .507  -.151  .114  -.225  -.352  -.376  .884  -.025  -.126  -.152  -.025  -.046  -.209  -.263  -.258  -.025  -.148  -.336 | .368  .173  .685  .106  .274  .153  -.132  .053  .444  .185  .465  .444  .416  .041  -.081  .286  .444  .056  -.423 | .759  -.212  -.223  .347  .042  .433  -.242  -.011  -.143  .851  -.143  -.143  -.156  .969  1.000  -.199  -.143  -.267  -.217 | 1.000  -.038  -.158  .408  .315  .422  -.537  .140  .162  .901  -.317  .162  .137  .856  .759  -.441  .162  -.048  -.280 |  |

|  | Resveratrol | Eriodictyol | Naringenin | **Pinobanksin-**  **3o-acetate** |
| --- | --- | --- | --- | --- |
| Acacetin Resveratrol Eriodictyol Naringenin  Pinobanksin-3o-acetate  (+)-Catechin Rutin lsorhamnetin Sakuranetin lsosakuranetin Daidzein Vitexin  Rosmarinic acid  Myricetin Ursolic acid Genistein  **Cynnamilidene acetic acid**  t-Cinnamic acid  Vanillin | -.038  1.000  .394  .787  .572  .418  -.358  .754  .819  -.308  -.212  .819  .809  -.265  -.212  -.294  .819  .025  -.321 | -.158  .394  1.000  .260  .338  .315  .241  -.036  .580  -.325  .722  .580  .567  -.280  -.223  .621  .580  -.089  -.338 | .408  .787  .260  1.000  .747  .807  -.349  .528  .783  .137  -.348  .783  .761  .261  .347  -.372  .783  -.186  -.388 | .315  .572  .338  .747  1.000  .604  -.352  .094  .812  -.107  -.275  .812  .796  -.026  .042  -.330  .812  -.070  .043 |

|  | (+) -Catechin | Rutin | lsorhamnetin | Sakuranetin |
| --- | --- | --- | --- | --- |
| Acacetin Resνeratrol Eriodictyol Naringenin  Pinobanksin-3o-acetate  (+)-Catechin Rutin lsorhamnetin Sakuranetin lsosakuranetin Daidzein Vitexin  Rosmarinic acid  Myricetin Ursolic acid Genistein  **Cynnamilidene acetic acid**  t-Cinnamic acid  Vanillin | .422  .418  .315  .807  .604  1.000  .123  .069  .661  .239  -.192  .661  .642  .359  .433  -.050  .661  -.171  -.466 | -.537  -.358  .241  -.349  -.352  .123  1.000  -.532  -.242  -.352  .468  -.242  -.248  -.303  -.242  .788  -.242  -.260  -.209 | .140  .754  -.036  .528  .094  .069  -.532  1.000  .332  .071  -.314  .332  .310  .027  -.011  -.437  .332  -.087  -.477 | .162  .819  .580  .783  .812  .661  -.242  .332  1.000  -.208  -.143  1.000  .997  -.179  -.143  -.199  1.000  .199  -.217 |

|  | **lsosakuraneti**  **η** | Daidzein | Vitexin | **Rosmarinic**  **acid** |  |
| --- | --- | --- | --- | --- | --- |
| Acacetin Resνeratrol Eriodictyol Naringenin  Pinobanksin-3o-acetate  (+)-Catechin Rutin lsorhamnetin Sakuranetin lsosakuranetin Daidzein Vitexin  Rosmarinic acid  Myricetin Ursolic acid Genistein  **Cynnamilidene acetic acid**  t-Cinnamic acid  Vanillin | .901  -.308  -.325  .137  -.107  .239  -.352  .071  -.208  1.000  -.208  -.208  -.227  .954  .851  -.289  -.208  -.081  -.316 | -.317  -.212  .722  -.348  -.275  -.192  .468  -.314  -.143  -.208  1.000  -.143  -.156  -.179  -.143  .909  -.143  -.267  -.217 | .162  .819  .580  .783  .812  .661  -.242  .332  1.000  -.208  -.143  1.000  .997  -.179  -.143  -.199  1.000  .199  -.217 | .137  .809  .567  .761  .796  .642  -.248  .310  .997  -.227  -.156  .997  1.000  -.195  -.156  -.216  .997  .267  -.159 |  |

|  | Myricetin | Ursolic acid | Genistein | **Cynnamiliden e acetic acid** |
| --- | --- | --- | --- | --- |
| Acacetin Resνeratrol Eriodictyol Naringenin  Pinobanksin-3o-acetate  (+)-Catechin Rutin lsorhamnetin Sakuranetin lsosakuranetin Daidzein Vitexin  Rosmarinic acid  Myricetin Ursolic acid Genistein  **Cynnamilidene acetic acid**  t-Cinnamic acid  Vanillin | .856  -.265  -.280  .261  -.026  .359  -.303  .027  -.179  .954  -.179  -.179  -.195  1.000  .969  -.249  -.179  -.190  -.272 | .759  -.212  -.223  .347  .042  .433  -.242  -.011  -.143  .851  -.143  -.143  -.156  .969  1.000  -.199  -.143  -.267  -.217 | -.441  -.294  .621  -.372  -.330  -.050  .788  -.437  -.199  -.289  .909  -.199  -.216  -.249  -.199  1.000  -.199  -.366  -.302 | .162  .819  .580  .783  .812  .661  -.242  .332  1.000  -.208  -.143  1.000  .997  -.179  -.143  -.199  1.000  .199  -.217 |

|  | **t-Cinnamic acid** | Vanillin |
| --- | --- | --- |
| Acacetin Resνeratrol Eriodictyol Naringenin  Pinobanksin-3o-acetate  (+)-Catechin Rutin lsorhamnetin Sakuranetin lsosakuranetin Daidzein Vitexin  Rosmarinic acid  Myricetin Ursolic acid Genistein  **Cynnamilidene acetic acid**  t-Cinnamic acid  Vanillin | -.048  .025  -.089  -.186  -.070  -.171  -.260  -.087  .199  -.081  -.267  .199  .267  -.190  -.267  -.366  .199  1.000  .489 | -.280  -.321  -.338  -.388  .043  -.466  -.209  -.477  -.217  -.316  -.217  -.217  -.159  -.272  -.217  -.302  -.217  .489  1.000 |
